# Clinical pharmacokinetics of potassium competitive acid blockers: a systematic review and meta-analysis

**DOI:** 10.3389/fphar.2025.1580969

**Published:** 2025-07-08

**Authors:** Jiaqi Liu, Jongsung Hahn

**Affiliations:** Department of Pharmacy, Jeonbuk National University, Jeonju, Jeollabuk, Republic of Korea

**Keywords:** pharmacokinetics, potassium competitive acid blockers, PCAB, meta-analysis, systematic review

## Abstract

**Background:**

Potassium competitive acid blockers (P-CABs) are a new class of acid suppressants that provide rapid and sustained inhibition of gastric acid secretion. Understanding the pharmacokinetic (PK) characteristics of P-CABs in various therapeutic uses is essential for optimizing treatment. This study aims to investigate the PK properties of P-CABs, focusing on drug interactions, food effects, and formulation impacts on their exposure and bioavailability.

**Methods:**

We systematically searched MEDLINE and Embase up to July 2024. The search terms included “Potassium competitive acid blockers” or “P-CABs” or “revaprazan” or “vonoprazan” or “tegoprazan” or “fexuprazan” or “keverprazan” or “zastaprazan” and “pharmacokinetics”.

**Results:**

A total of 37 studies were included. Meta-analysis and qualitative studies indicated that clarithromycin significantly increased vonoprazan and tegoprazan exposure [geometric mean ratio (GMR) (90% confidence interval (CI))] of AUC and Cmax: 1.565 (1.443, 1.687) and 1.538 (1.454, 1.621) for vonoprazan, 2.624 (2.513, 2.735) and 1.876 (1.771, 1.981) for tegoprazan, respectively. Vonoprazan had more of an inhibitory effect on cytochrome P450 (CYP) 3A and CYP2C19 compared to tegoprazan. P-CABs showed minimal interactions with nonsteroidal anti-inflammatory drugs or aspirin and were largely unaffected by food intake, except keverprazan and zastaprazan, which showed increased exposure.

**Discussion:**

It is important to select the appropriate P-CABs by considering the degree of influence on CYP enzymes, the dosage form, and food interactions. Studies on the interaction between P-CABs and antibiotics used to treat *H. pylori* infections, such as metronidazole, tetracycline, levofloxacin, or rifabutin, as well as non-vitamin K antagonist oral anticoagulants are lacking, and further research is needed.

## 1 Introduction

Decreasing stomach acid production is crucial in the treatment of gastroesophageal reflux disease (GERD) and peptic ulcer disease (PUD). The Montreal Protocol defines GERD as the regurgitation of the stomach contents into the esophagus, resulting in discomfort or complications (Vakil et al., 2006). The pooled prevalence of GERD symptoms reported at least once a week in population-based studies worldwide is approximately 13%, but there is considerable geographic variation (Eusebi et al., 2018). *Helicobacter pylori* (*H. pylori*) is a prevalent bacterial infection that can cause PUD and lead to complications such as gastric mucosa-associated lymphoid tissue lymphoma and gastric cancer (Marshall and Warren, 1984).

Proton pump inhibitors (PPIs) and H2-receptor antagonists (H2RAs) are used for GERD and PUD. Despite having more potent and sustained acid inhibition compared with H2RAs, PPIs have some limitations (Katz et al., 2022). Being a prodrug, PPIs require activation in an acidic environment to form irreversible covalent bonds with cysteine residues on the H+/K + ATPase. This activation mechanism necessitates taking PPIs 30–60 min before meal to ensure optimal acid suppression. Furthermore, PPIs are primarily metabolized by cytochrome P450 (CYP) 2C19 enzyme, and genotypic variation can affect their efficacy in individuals (Katz et al., 2006). Studies have shown that up to 40% of patients with GERD do not experience adequate symptom relief with standard once-daily PPIs doses (Cicala et al., 2013). Moreover, PPIs have the issue of nocturnal acid breakthrough, which might be related to their relatively short half-life of approximately 1 h. Additionally, the time to reach maximum plasma concentration (Tmax) of PPIs varies significantly from 1 to 5 h, which is influenced by the formulation or food (Shin and Kim, 2013).

Potassium competitive acid blockers (P-CABs) are a new class of acid suppressants that inhibit the H+/K + -ATPase enzyme through reversible and non-covalent binding to active or inactive potassium-binding sites. Unlike traditional PPIs, P-CABs do not require activation in an acidic environment, can be taken regardless of dietary intake, have a longer half-life, and are metabolized primarily through CYP3A4 (Ant et al., 2024; Ganoci et al., 2017; Daly, 2015). Therefore, P-CABs have several advantages over PPIs, including a faster onset of effect, longer duration of acid inhibition, and less variation between individuals. Six P-CABs have been approved and marketed, including revaprazan (Korea, approved in 2005), vonoprazan (Japan, approved in 2014), tegoprazan (Korea, approved in 2018), fexuprazan (Korea, approved in 2021), keverprazan (China, approved in 2023), and zastaprazan (Korea, approved in 2024).

Recent systematic reviews and meta-analyses have demonstrated that P-CABs offer certain benefits in the treatment of acid-related diseases over PPIs, particularly in H. pylori eradication, GERD, and peptic ulcer (Kanu and Soldera, 2024; Seo et al., 2024; Wang et al., 2024). one meta-analysis has shown that vonoprazan is superior to traditional PPIs in clarithromycin-resistant *H. pylori* eradication, without increasing adverse effects (Yamade et al., 2017). Additionally, vonoprazan proved more effective than PPIs in both short-term and long-term management of severe cases of erosive esophagitis (Fang et al., 2024). Vonoprazan and tegoprazan performed better than PPIs and H2RAs for nocturnal acid suppression (Zou et al., 2024).

Previous studies primarily focused only on the efficacy of P-CABs. However, no systematic review that covers all clinical PK parameters, as well as its drugs or foods interactions and ethnicity and formulation effect has been reported till now. Therefore, this study aims to summarize the PK characteristics of P-CABs across various therapeutic uses.

## 2 Methods

### 2.1 Search strategy

The Preferred Reporting Items for Systematic Reviews and Meta-Analyses (PRISMA) 2020 checklist was used as a guide to perform, complete, and report the review. We searched studies including PK parameters of P-CABs on Medline (via PubMed) and Embase from database inception to search date of July 2024. To complete the searches, we used keywords and phrases as follows: P-CABs (“Potassium competitive acid blockers”, “P-CABs”, “revaprazan”, “vonoprazan”, “tegoprazan”, “fexuprazan”, “keverprazan”, “zastaprazan”) in combinations with “pharmacokinetics”. Articles were included if they reported available PK parameters. However, we excluded studies written in a language other than English, *in vitro* studies, and animal studies. We also excluded PK clinical trial studies for the purpose of marketing authorization of drugs, studies simply describing PK parameters, such as simple dose ascending studies, and studies that conducted PK modeling based on previously published data.

### 2.2 Data extraction and quality assessment

The general information of the study (author, publication year, country), patient characteristic (sex, age, comorbidity), study design (number of participants, dosing regimen), and PK parameters were extracted independently by two researchers (JH and JQ).

### 2.3 Data analysis

All meta-analyses were conducted using the R software (version 4.3.1). In order to determine degree of PK change in P-CABs owing to interactions with drugs or foods, the Cmax and AUC values were evaluated using the geometric mean ratio (GMR) [combination administration/single administration or fed state/fasted state] and 90% confidence interval (CI). If the 90% CI of Cmax and AUC fell completely between 0.80 and 1.25, there was no drug or foods interaction. For the time to Tmax and t1/2 and other parameters, the standardized mean difference (SMD) along with its 95% CI was used for assessment. If the 95% CI of the SMD did not include 0, it indicated statistically significant difference between the parameters. When Tmax was expressed as the median, along with its corresponding minimum and maximum values, it was converted to the mean and standard deviation (SD) using the method described by Hozo et al. (Hozo et al., 2005). Parametric values of mean and coefficient of variation (CV) were converted to SD for statistical convenience. Common or random model for meta-analysis was used based on the heterogeneity level in treatment effects across included studies.

## 3 Results

### 3.1 Study description

**FIGURE 1:**
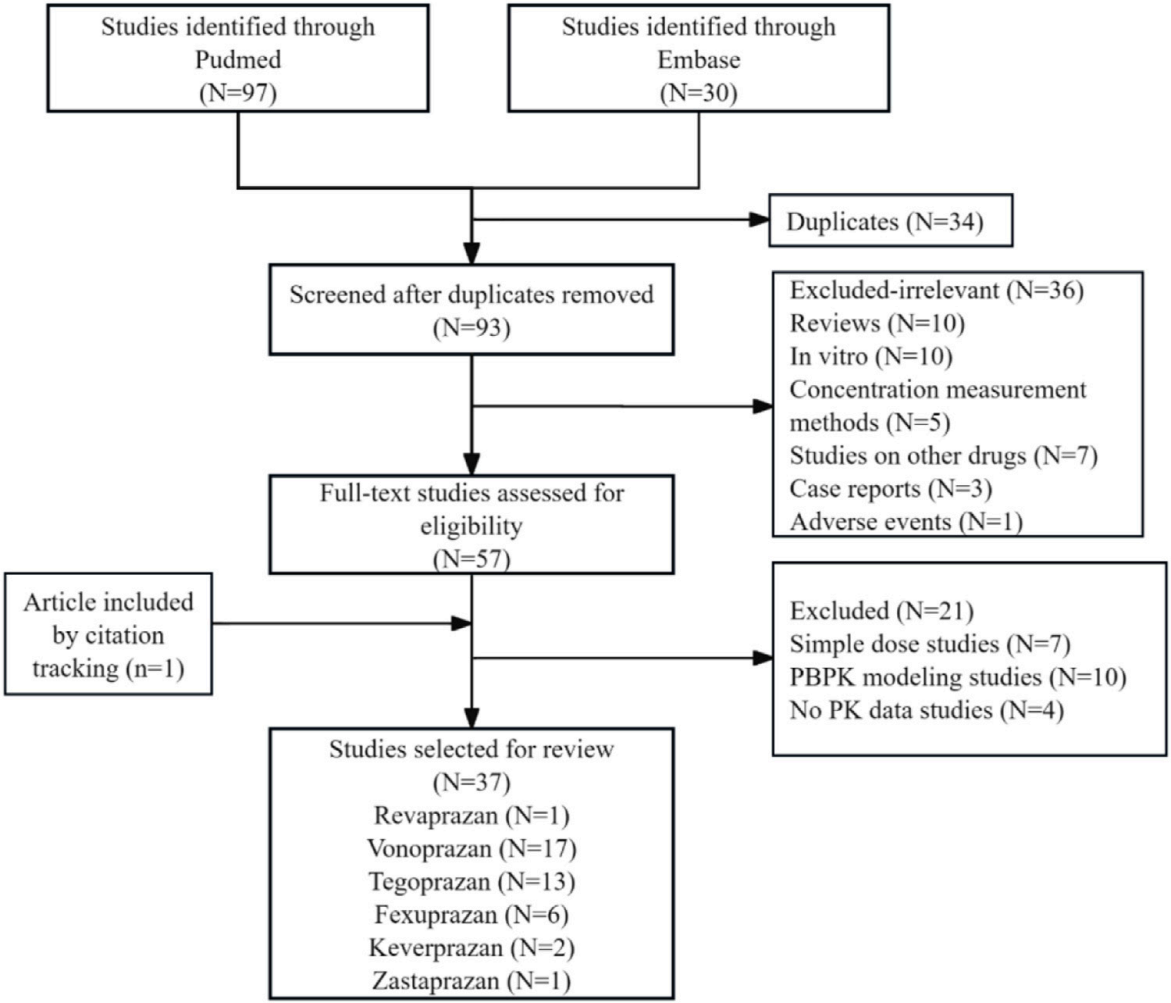
Flow diagram for selection and inclusion of the studies. Three of the included articles were duplicates (two studies on vonoprazan and tegoprazan 2; one study on vonoprazan and keverprazan).

The literature was systematically screened based on the inclusion and exclusion criteria (Figure 1). After removing duplicates, 93 records were evaluated by titles and abstracts; 36 studies were excluded as irrelevant, including reviews, *in vitro* studies, case reports, and studies on concentration measurement methods, other drugs, or adverse events. Full-text assessment of 57 articles excluded 21 studies: 7 simple dose ascending studies, 10 physiologically based pharmacokinetic (PBPK) modeling, and 4 studies not reporting any PK data, while one additional article was included through citation tracing. Ultimately, 37 studies were selected for review: revaprazan (1 study), vonoprazan (17 studies), tegoprazan (13 studies), fexuprazan (6 studies), keverprazan (2 studies), and zastaprazan (1 study). Vonoprazan and tegoprazan shared two articles, and vonoprazan and keverprazan shared one article. Detailed study characteristics are presented in Supplementary Table S1.

### 3.2 Drug interaction

#### 3.2.1 *H. pylori* infection treatment

**FIGURE 2:**
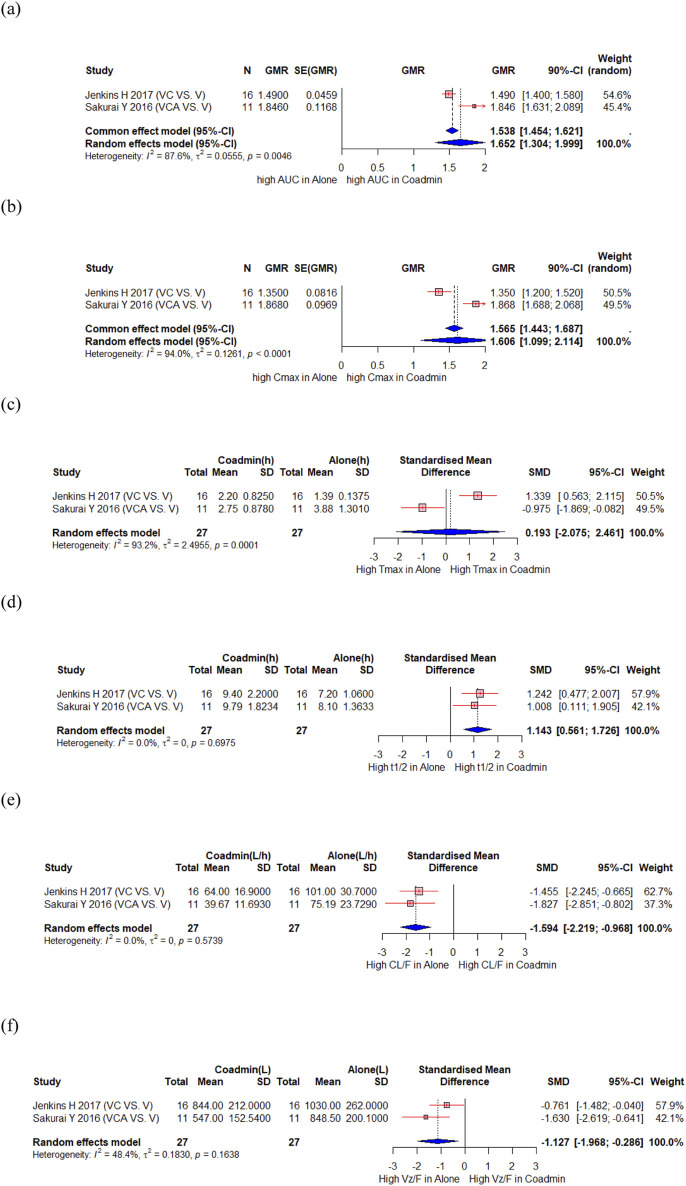
Forest plot showing changes in PK parameters of vonoprazan when administered alone and co-administered with others drug. **(a)** GMR of AUC. **(b)** GMR of Cmax. **(c)** SMD of Tmax. **(d)** SMD of t1/2. **(e)** SMD of CL. **(f)** SMD of Vd. AUC, area under the concentration curve; CL/F, apparent clearance; Cmax, peak concentration; t1/2, elimination half-life; Tmax, time to reach Cmax; Vz/F, apparent volume of distribution; CI, confidence interval; GMR, geometric mean ratio; SD, standard deviation; SE, standard error; SMD, standard mean difference; Coadmin: coadministration; V, vonoprazan; VC, vonoprazan and clarithromycin; VCA, vonoprazan and clarithromycin and amoxicillin.

Jenkins H et al. conducted a sequential study in which subjects received vonoprazan on days 1 and 8 and clarithromycin on days 3–9 to assess the influence of clarithromycin on the PK of vonoprazan (Jenkins et al., 2017). Sakurai Y et al. compared PK of vonoprazan when used as monotherapy and triple therapy (vonoprazan/clarithromycin/amoxicillin (Sakurai et al., 2016). A total of 27 participants from these 2 studies were included in the meta-analysis, which showed significant changes in the AUC and Cmax for vonoprazan, with a GMR (90% CI) of 1.652 (1.304, 1.999) and 1.606 (1.099, 2.114), respectively (Figures 2a,b). However, the Tmax of vonoprazan did not change significantly, with an SMD (95% CI) of 0.193 (−2.075, 2.461) (Figure 2c). Moreover, there was a significant increase in t1/2, with an SMD (95% CI) of 1.143 (0.561, 1.726) (Figure 2d). Changes in these PK parameters resulted in significantly lower clearance (CL) and volume of distribution values with SMD (90% CI) of −1.594 (−2.219, −0.968) and −1.127 (−1.968, −0.286), respectively (Figures 2e,f). There was significant decrease in the AUC and Cmax for the vonoprazan metabolites M-I, M-II, and M-III, with a GMR (90% CI) of 0.771 (0.708, 0.834) and 0.624 (0.497, 0.751) for M-I; 0.518 (0.459, 0.577) and 0.488 (0.426, 0.551) for M-II; 0.686 (0.167, 1.205) and 0.520 (0.079, 0.961) for M-III, respectively. However, the AUC and Cmax of M-IV-Sul, a metabolite of vonoprazan, significantly increased, with a GMR (90% CI) of 2.112 (1.628, 2.596) and 1.853 (1.149, 2.558), respectively (Supplementary Figure S1). Sakurai Y et al. also reported no notable PK interactions between vonoprazan and metronidazole or amoxicillin in triple therapy of vonoprazan/metronidazole/amoxicillin.

In two studies, the PK interaction of vonoprazan with bismuth was compared with that of PPI with bismuth. (Huh et al., 2022; Miao et al., 2023). Huh KY et al. compared the effects of vonoprazan and lansoprazole on bismuth PK, and as a result, the GMRs of AUC and Cmax (vonoprazan to lansoprazole) were 0.94 (0.71, 1.23) and 1.05 (0.72, 1.54), respectively (Huh et al., 2022). Miao J et al. compared vonoprazan and esomprazole, and the results showed that the GMRs of AUC and Cmax (vonoprazan to esomeprazole) were 1.07 (0.82, 1.40) and 1.30 (0.94, 1.81), respectively. (Miao et al., 2023).

**FIGURE 3:**
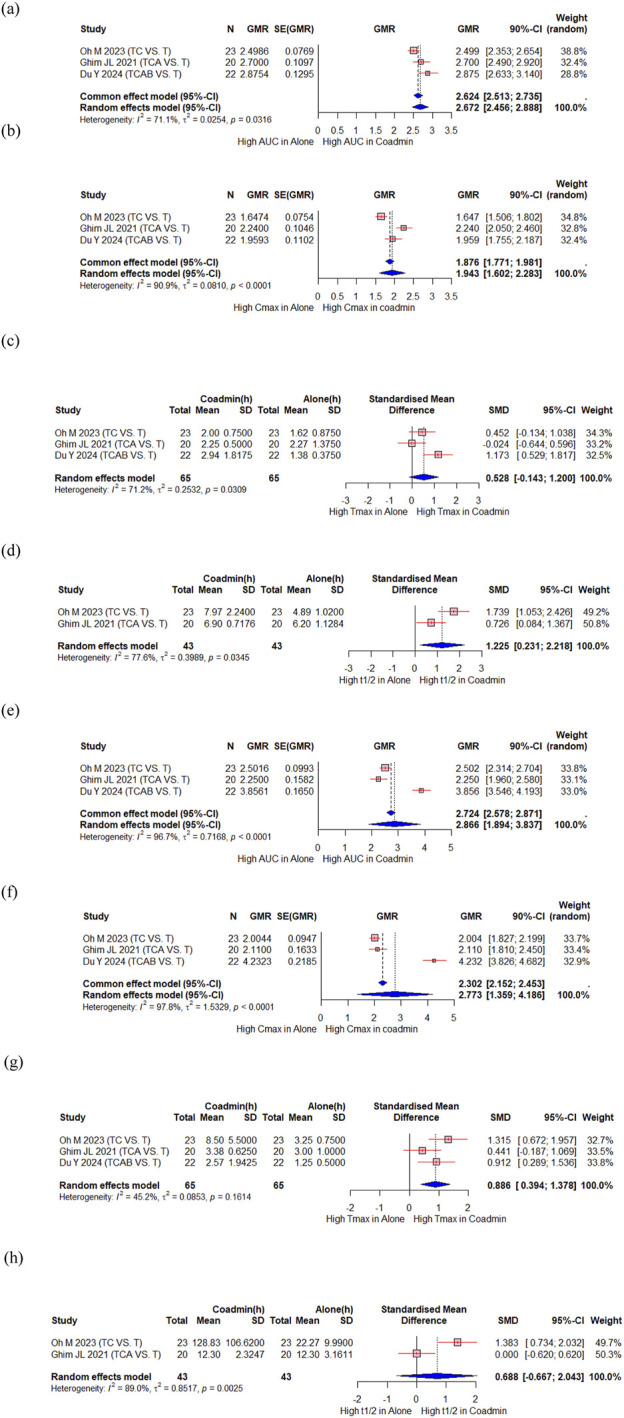
Forest plot showing changes in PK parameters of tegoprazan and its metabolite M1 when administered alone and co-administered with others drug. **(a)** GMR of AUC of tegoprazan. **(b)** GMR of Cmax of tegoprazan. **(c)** SMD of Tmax of tegoprazan. **(d)** SMD of t1/2 of tegoprazan. **(e)** GMR of AUC of M1. **(f)** GMR of Cmax of M1. **(g)** SMD of Tmax of M1. **(h)** SMD of t1/2 of M1. AUC, area under the concentration curve; Cmax, peak concentration; t1/2, elimination half-life; Tmax, time to reach Cmax; CI, confidence interval; GMR, geometric mean ratio; SD, standard deviation; SE, standard error; SMD, standard mean difference; Co-admin: coadministration; T, tegoprazan; TC, tegoprazan and clarithromycin; TCA, tegoprazan and clarithromycin and amoxicillin; TCAB, tegoprazan and clarithromycin and amoxicillin and bismuth.

Oh M et al. evaluated the PK interaction between tegoprazan and clarithromycin (Oh M. et al., 2023), Ghim JL et al. evaluated the PK interaction between tegoprazan and clarithromycin/amoxicillin (Ghim et al., 2021), and Du Y et al. evaluated the PK interaction between tegoprazan and clarithromycin/amoxicillin/bismuth (Du et al., 2024). A total of 65 participants from the three studies were included in the meta-analysis. There were significant increase in the AUC and Cmax of tegoprazan, with a GMR (90% CI) of 2.672 (2.456, 2.888) and 1.943 (1.602, 2.283), respectively (Figures 3a,b). The Tmax of tegoprazan was unchanged with an SMD (95% CI) of 0.528 (−0.143, 1.200) (Figure 3c), and t1/2 increased significantly with an SMD (95% CI) of 1.225 (0.231, 2.218) (Figure 3d). There were significant changes in the AUC and Cmax for M1, an active metabolite of tegoprazan, with a GMR (90% CI) of 2.866 (1.894, 3.837) and 2.773 (1.359, 4.186), respectively (Figures 3e,f). The Tmax of tegoprazan M1 significantly increased, with an SMD (95% CI) of 0.886 (0.394, 1.378) (Figure 3g). However, the t1/2 of tegoprazan M1 did not change significantly, with an SMD (95% CI) of 0.688 (−0.667, 2.043) (Figure 3h).

Above three studies described clarithromycin PK changes when co-administered with tegoprazan. There is a slight change in the AUC of clarithromycin with a GMR (90% CI) of 1.188 (1.073, 1.303), whereas the Cmax remained stable, showing a GMR (90% CI) of 0.986 (0.809, 1.163). Additionally, there was significant change in the PK of 14-OH-clarithromycin, an active metabolite of clarithromycin, with a GMR (90% CI) of 1.750 (1.446, 2.054) and 1.600 (1.254, 1.945), for AUC and Cmax, respectively, and an SMD (95% CI) of 0.948 (0.278, 1.617) for Tmax. However, the t1/2 did not change significantly, with an SMD (95% CI) of 0.010 (−0.531, 0.552) (Supplementary Figure S2).

The PK parameters of amoxicillin when co-administered with tegoprazan changed with a GMR (90% CI) of 1.050 (0.838, 1.263) and 0.875 (0.639, 1.111) for AUC and Cmax, respectively and an SMD (95% CI) of 0.636 (0.158, 1.114) for Tmax (Supplementary Figure S3). The effects of tegoprazan on bismuth PK showed significant increases in the AUC and Cmax of bismuth with a GMR (90% CI) of 1.795 (1.522, 2.069) and 1.559 (1.210, 1.907), respectively. (Supplementary Figure S3). Jeon JY et al. evaluated the PK interaction between tegoprazan with metronidazole, tetracycline, and bismuth, and the results showed that systemic exposure to tegoprazan, tegoprazan M1, and tetracycline decreased, while exposure to bismuth increased. (Jeon et al., 2021).

#### 3.2.2 NSAIDs/aspirin

Sakurai Y et al. evaluated the PK interaction between vonoprazan and low-dose aspirin or commonly used nonsteroidal anti-inflammatory drugs (NSAIDs), namely, loxoprofen, diclofenac, and meloxicam (Sakurai et al., 2017). The results showed that the 90% CIs of the ratios of least square means were within the range (0.8, 1.25) for C max and AUCs of vonoprazan except that the lower bound for Cmax was 0.695 when co-administered with loxoprofen. Effect of vonoprazan on the pharmacokinetics of aspirin was significant with 56% increase in Cmax and 23% increase in AUC0-48 of aspirin. However, due to the large observed inter-subject variability and little difference observed in the pharmacokinetics of salicylic acid, active metabolite, it was considered to have no clinically relevant drug–drug interactions.

Moon SJ et al. evaluated the PK interaction between tegoprazan and NSAIDS (Moon et al., 2022). The results showed that the 90% CIs of the geometric least squares mean was within the range (0.8, 1.25) for C max and AUCs of tegoprazan and naproxen. Meanwhile, Cmax for aceclofenac and celecoxib were 1.31 (1.08, 1.60) and 1.18 (0.97, 1.43) when co-administered with tegoprazan, respectively. Since the AUC was unchanged in all three NSAIDs when co-administered with tegoprazan, increase of Cmax in aceclofenac and celecoxib would not be clinically significant in practice.

Oh J et al. evaluated the PK interaction between fexuprazan and aspirin (Oh J. et al., 2023). The study found that the 90% CIs of the GMR were within the range (0.8, 1.25) for systemic exposure of aspirin and salicylic acid when co-administered with fexuprazan. The systemic exposure of fexuprazan was decreased up to 20%, which was not regarded as clinically meaningful considering the previously reported exposure–response relationship. Won H et al. evaluated the PK interactions between fexuprazan and NSAIDs (Won et al., 2024). The study showed that 90% CIs of the GMR were within the range (0.8, 1.25) for systemic exposure of fexuprazan when co-administered with celecoxib or meloxicam. However, when administered in combination with naproxen, the differences in PK parameters of fexuprazan were observed with a GMR (90% CI) of 1.22 (1.02, 1.46) and 1.19 (1.00, 1.43) for Cmax and AUCτ, respectively. Won H et al. explained that previous studies have demonstrated the safety and tolerability of fexuprazan up to 160 mg for 7 days, a slight increase in systemic exposure at 40 mg twice-daily doses of fexuprazan by naproxen in this study is within the safe range.

#### 3.2.3 CYP substrate

**FIGURE 4:**
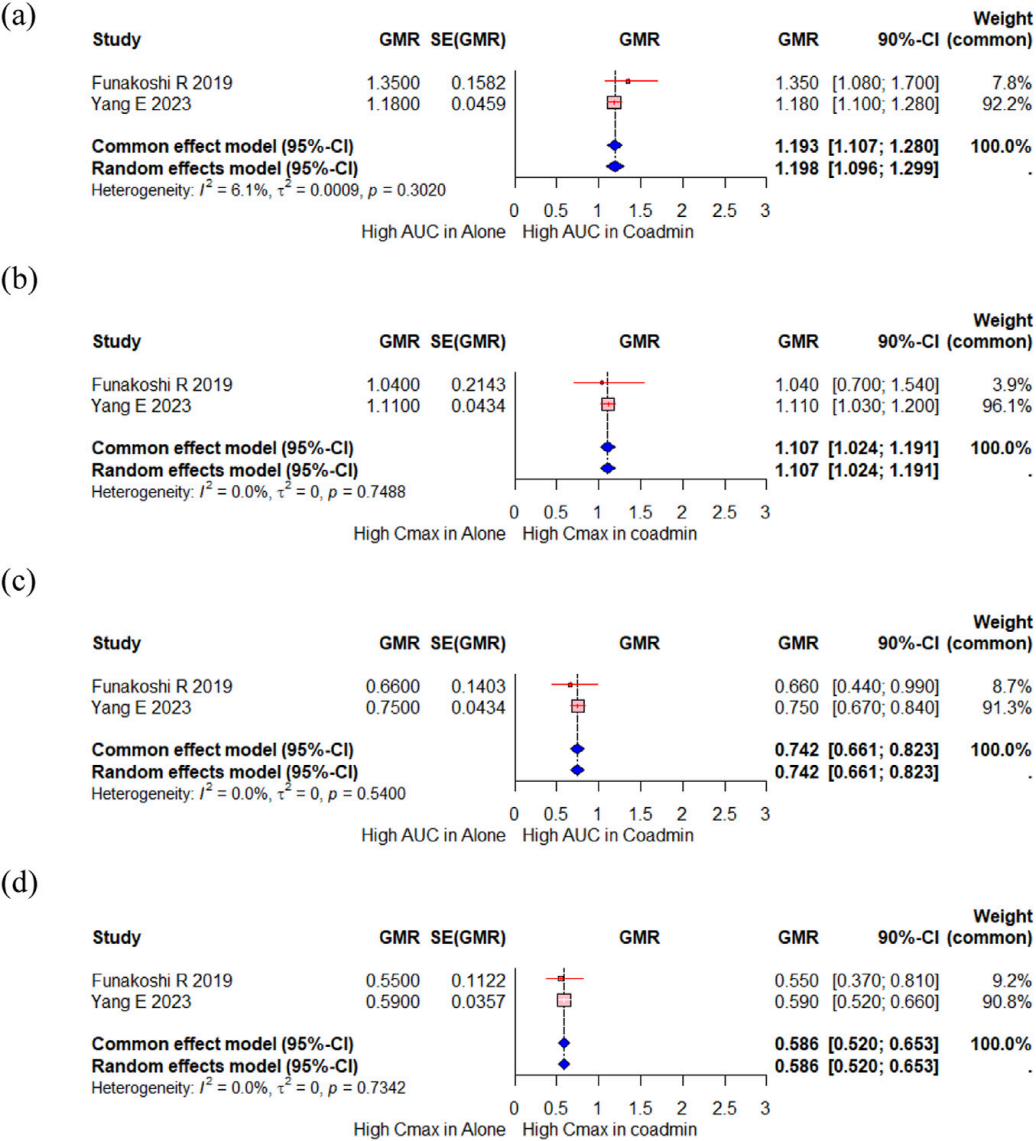
Forest plot showing changes in PK parameters of proguanil and cycloguanil when administered alone and co-administered with vonoprazan. **(a)** GMR of AUC of proguanil. **(b)** GMR of Cmax of proguanil. **(c)** GMR of AUC of cycloguanil. **(d)** GMR of Cmax of cycloguanil. AUC, area under the concentration curve; Cmax, peak concentration; CI, confidence interval; GMR, geometric mean ratio; SD, standard deviation; SE, standard error; SMD, standard mean difference; Coadmin: coadministration.

A total of 23 participants from two studies investigated changes in the PK of proguanil, a CYP2C19 substrate, when administered with vonoprazan compared to when administered alone (Funakoshi et al., 2019; Yang E. et al., 2023). In the meta-analysis, there were slight changes in the AUC for proguanil, with a GMR (90% CI) of 1.193 (1.107, 1.280) (Figure 4a). The Cmax of proguanil did not change significantly, with a GMR (90% CI) of 1.107 (1.024,1.191) (Figure 4b). However, there were significant changes in the AUC and Cmax for cycloguanil, an active metabolite of proguanil, with a GMR (90% CI) of 0.742 (0.661, 0.823) and 0.586 (0.520, 0.653), respectively (Figures 4c,d). Yang E et al. compared the PK change of proguanil among tegoprazan, vonoprazan and esomeprazole (Yang E. et al., 2023). No changes in the systemic exposure of proguanil and cycloguanil was observed when proguanil was co-administered with tegoprazan; however, the systemic exposure of proguanil increased and that of cycloguanil decreased when proguanil was co-administered with vonoprazan or esomeprazole, and magnitude of this change was greater with esomeprazole.

Hwang S et al. evaluated changes in the PK of atorvastatin, a CYP3A4 substrate, when co-administered with vonoprazan compared to tegoprazan (Hwang et al., 2021). The result showed that the systemic exposure of atorvastatin increased when co-administered with vonoprazan, with a GMR (90% CI) of 1.17 (1.04, 1.32) for Cmax and 1.28 (1.22, 1.34) for AUC. Similarly, the systemic exposure of atorvastatin lactone, an inactive lactone form of atorvastatin, increased with a GMR (90% CI) of 1.32 (1.24, 1.41) for Cmax and 1.29 (1.23, 1.35) for AUC. Although the Cmax of 2-hydroxyatorvastatin, an active metabolite of atorvastatin, decreased with a GMR (90% CI) of 0.7 (0.63, 0.78), there was no significant difference in its AUC, with a GMR (90% CI) of 0.91 (0.87, 0.95). Hwang S et al. explained this by the fact that vonoprazan with high luminal concentrations might inhibit the intestinal CYP3A4, thus increasing the systemic exposure of orally administered atorvastatin. However, they found no changes in the systemic exposure of atorvastatin, 2-hydroxyatorvastatin, and atorvastatin lactone when atorvastatin was co-administered with tegoprazan.

Mulford DJ et al. evaluated the impact of vonoprazan on the exposure of oral midazolam, an index substrate for CYP3A (Mulford et al., 2023). The results showed that when midazolam was co-administered with vonoprazan, the systemic exposure of midazolam increased, with a GMR of 1.93 (1.61, 2.33) for Cmax and 1.89 (1.51, 2.37) for AUC, and that of 1-hydroxymidazolam, an active metabolite of midazolam, increased by 1.25 (0.98, 1.59) for Cmax and 1.31 (1.31, 1.67) for AUC. The elimination of midazolam and 1-hydroxymidazolam was comparable when midazolam was administered either alone or co-administered with vonoprazan, as reflected by no considerable changes in its t1/2.

Two studies retrospectively compared the concentrations of tacrolimus when switching from rabeprazole to vonoprazan in renal transplant recipients (Mei et al., 2020; Watari et al., 2021). Mei et al. demonstrated that the mean ± SD of tacrolimus trough concentration/tacrolimus dose (ng/mL)/(mg/day) increased from 1.98 ± 1.02 to 2.19 ± 1.15 (*p* < 0.001) after the conversion from rabeprazole to vonoprazan. Liver enzymes and estimated glomerular filtration rate were significantly elevated within normal ranges. However, Watari S et al. reported no statistically significant differences in tacrolimus trough levels after the conversion from rabeprazole to vonoprazan.

#### 3.2.4 Revaprazan with itopride

Choi HY et al. assessed changes in the PK between revaprazan and itopride (Choi et al., 2012). The study results showed that the GMR (90% CI) of Cmax and AUCτ for revaprazan and itopride were within the bioequivalence interval – 0.92 (0.84, 1.00) and 0.96 (0.89, 1.03), respectively for revaprazan and 1.07 (0.96, 1.20) and 1.12 (1.06, 1.18), respectively for itopride. There were no significant changes in the CL and Tmax values for revaprazan and itopride.

#### 3.2.5 Vonoprazan with osimertinib

Yokota H et al. conduct univariate analysis to examine factors affecting osimertinib plasma levels in patients with non-small cell lung cancer (NSCLC) (Yokota et al., 2022). No acid suppressant group had an AUC0-24 of 6,350 ng*h/mL and a Ctrough of 173 ng/mL; the H2RAs or esomeprazole group had an AUC0-24 of 8,305 ng*h/mL and a Ctrough of 241 ng/mL, and the vonoprazan group had an AUC0-24 of 9,669 ng*h/mL and a Ctrough of 331 ng/mL, which showed statistically significant differences (*p* = 0.021 for AUC0-24 and *p* = 0.046 for Ctrough). Vonoprazan increased osimertinib absorption by inhibiting P-glycoprotein (P-gp) activity, thus elevating its plasma concentration. However, in multivariate analysis, its influence was negligible.

### 3.3 Effect of food on P-CABs

Mulford DJ et al. described PK changes of vonoprazan under fasted and high-fat meal conditions (Mulfo et al., 2022). The study found that food did not significantly affect PK parameters with a GMR (90% CI) for Cmax, AUC0-24, and AUCinf being 1.05 (0.98, 1.12), 1.13 (1.09, 1.18), and 1.15 (1.11, 1.19), respectively. Additionally, the median Tmax increased from 2 h (0.75, 4.00) in the fasted state to 4 h (1.98, 6.02) in the fed state.

Two studies described PK changes of tegoprazan under fasted and fed states (Han et al., 2021; Yoon et al., 2021). Han S et al. focused on comparing the PK profile of tegoprazan between standard meals and fasting, whereas Yoon DY et al. compared high-fat diet with fasting. In the standard diet, tegoprazan Cmax significantly decreased (GMR 0.5379, 90% CI: 0.5000, 0.5786), Tmax increased from 1 h in the fasted state to 3.92 h in the fed state, and AUC remained unchanged (GMR 0.9016, 90% CI: 0.8687, 0.9358). M1 showed a decreased Cmax (GMR 0.5679, 90% CI: 0.5180, 0.6227) and extended Tmax from 3.92 h in the fasted state to 7.94 h in the fed state, with a slight decrease in AUC (GMR 0.8058, 90% CI: 0.7692, 0.8441) (Han et al., 2021). Under high-fat diet condition, tegoprazan Cmax significantly decreased (GMR 0.5396, 90% CI: 0.4211, 0.6914), Tmax increased from 1 h in the fasted state to 3 h in the fed state, whereas AUC remained stable (GMR 1.0455, 90% CI: 0.9661, 1.1316). M1 showed reduced Cmax and AUC (GMR 0.7214, 90% CI: 0.6314, 0.8242 and 0.8432, 90% CI: 0.7815, 0.9099, respectively), with Tmax stable at 8 h (Yoon et al., 2021).

Sunwoo J et al. examined the PK alterations of fexuprazan in the fed state compared with the fasted state (Sunwoo et al., 2018). The study indicated that eating food before drug administration did not result in clinically significant impact on the PK of fexuprazan. The GMR of the fed state to the fasted state for Cmax and AUC were 1.06 and 1.09, respectively.

Zhou S et al. evaluated the impact of high-fat meal on keverprazan PK (Zhou et al., 2023). The study results showed that intake of high-fat meal increased keverprazan exposure levels, with a GMR (90% CI) of 1.268 (1.090, 1.475) and 1.349 (1.238, 1.469) for Cmax and AUCinf, respectively. However, the Tmax of keverprazan was comparable between the two groups, with a median (min, max) of 1.50 (1.00, 4.00) h in the fasted state and 1.75 (1.25, 6.01) h in the fed state. The GMR values of Cmax for the metabolite M9 were reduced 35%, and Tmax was delayed by approximately 1 h. However, there was no obvious effect of food on t1/2.

Hwang I et al. showed that a high-fat meal decreased the peak zastaprazan plasma level (mean (SD)) (45.65 (9.15) μg/L in fed vs. 101.14 (26.49) μg/L in fasted) while increased the overall systemic exposure (439.27 (212.90) μg*h/L in fed vs. 359.15 (177.18) μg*h/L in fasted) and prolonged Tmax (mean (min-max)) (4.00 (0.75, 6.00) h in fed vs. 0.75 (0.50, 0.75) h in fasted) of zastaprazan (Hwang et al., 2023).

### 3.4 Effect of ethnicity on P-CAB

Two studies described PK differences of vonoprazan in Japanese and non-Japanese populations (Jenkins et al., 2015; Sakurai et al., 2015). Both studies used power models with fixed effects for the variable of region (Japanese vs. non-Japanese), and the result showed that no potential regional differences between Japanese and non-Japanese patients.

Hwang JG et al. compared the pharmacodynamics (PD), PK, and safety of fexuprazan among Korean, Caucasian, and Japanese populations (Hwang et al., 2020). The results showed that the differences in the systemic exposure of fexuprazan between Caucasians and Koreans and between Japanese and Koreans after single and multiple doses were not statistically significant (all *p* > 0.05). The fraction excreted in the urine was also comparable among the three ethnicities.

### 3.5 Effect of formulation/routes on P-CAB

Hwang JG et al. compared the PK of two formulations (conventional vs. one with improved productivity and stability) of tegoprazan 100 mg tablets (Hwang et al., 2019). The results demonstrated that the PK parameters of the two tegoprazan formulations were comparable, with GMR (90% CI) for Cmax and AUClast of tegoprazan were 0.98 (0.85, 1.12) and 1.03 (0.93, 1.13), respectively, and that of M1 were 0.99 (0.89, 1.11) and 1.01 (0.93, 1.09), respectively. Lee JA et al. compared the PK between the conventional tablet and orally disintegrating tablet (ODT) of 50 mg tegoprazan (Lee et al., 2023). The results demonstrated that the PK profiles were equivalent, with GMR (90% CI) for AUClast, Cmax, and AUCinf of tegoprazan were 0.9291 (0.8873, 0.9729), 0.9680 (0.8865, 1.0569), and 0.9255 (0.8835, 0.9695), respectively for ODT with water to the conventional tablet, and 0.9963 (0.9169, 1.0127), 1.0387 (0.9569, 1.1276), and 0.9637 (0.9166, 1.0131), respectively for ODT without water to the conventional tablet. The GMR (90% CI) for AUClast, Cmax, and AUCinf of M1 were 0.9963 (0.9552, 1.0391), 1.0687 (1.0069, 1.1343), and 0.9993 (0.9487, 1.0527), respectively for ODT with water to the conventional tablet, and 1.0005 (0.8914, 1.1230), 0.9850 (0.8866, 1.0943), and 1.0022 (0.9002, 1.1157), respectively for ODT without water to the conventional tablet. Park S et al. explored the PK and PD of various combinations of tegoprazan with immediate-release (IR) and delayed-release (DR) formulations (Park et al., 2023). The PK results indicated that tegoprazan absorption delayed and Cmax decreased as the DR to IR ratio increased compared with that when IR was administered alone. When tegoprazan was administered in a 1:1 ratio of IR and DR, the 50 and 100 mg dose groups had the most similar AUClast, with a GMR (90% CI) for AUClast of tegoprazan IR 25 mg plus DR 25 mg to the IR 50 mg was 0.98 (0.82, 1.17), and 0.93 (0.81, 1.05) for tegoprazan IR 50 mg plus DR 50 mg to the IR 100 mg. According to the PK results, they concluded that the 1:1 ratio formulation of IR and DR may be a suitable alternative to conventional IR formulations to maintain initial action while maintaining gastric inhibition for 24 h and at night. Kim HS et al. evaluated and compared the PK of tegoprazan ODT administered via nasogastric tube with oral administration (Kim et al., 2024). The PK results indicated that tegoprazan was rapidly absorbed when administered orally or via nasogastric tube, with median Tmax of 0.75 h and 0.5 h, respectively. The GMR (90% CI) for Cmax and AUCt of tegoprazan when administered via nasogastric tube compared with oral administration was 1.1087 (1.0243, 1.2000) and 1.0023 (0.9620, 1.0442), respectively, and fell within the bioequivalence range.

Shin W et al. conducted bioequivalence test of a new dosage form of four 10 mg tablet to a previously approved 40 mg tablet of fexuprazan (Shin et al., 2023). The results demonstrated that the PK of four fexuprazan 10 mg tablets were comparable to one fexuprazan 40 mg tablet, with a GMR (90% CI) for Cmax and AUClast of the four fexuprazan 10 mg tablets to one fexuprazan 40 mg tablet were 1.0290 (0.9352, 1.1321) and 1.0290 (0.9476, 1.1174), respectively, meeting the bioequivalence criteria of 0.8–1.25. Yang AY et al. compared the safety and PK of a previous approved tablet with size-reduced 20 mg tablet of fexuprazan (Yang A. Y. et al., 2023). The results demonstrated that the PK profile of size-reduced fexuprazan was comparable to a previous formulation of fexuprazan, with a GMR (90% CI) of 1.1014 (0.9892, 1.2265) and 1.0530 (0.9611, 1.1536) for Cmax and AUClast, respectively.

## 4 Discussion

P-CABs are widely used for GERD and PUD treatment. Their PK properties can be influenced by drug and food interactions, ethnicity, drug formulation, and administration route under various conditions. This review combines existing clinical data to evaluate PK changes of P-CABs under different administration conditions.

The 2022 Maastricht VI/Florence Consensus report highlighted that P-CABs based treatment regimens are superior or not inferior to conventional PPI-based triple therapies. The consensus also concluded that P-CABs are particularly effective for patients with antimicrobial-resistant *H. pylori* infections, which is an important consideration given the growing global concern about antibiotic resistance (Patel et al., 2024).

Clarithromycin was used as triple or quadruple *H. pylori* eradication therapy. Clarithromycin, a CYP3A4 inhibitor, shows inhibitory effect on vonoprazan metabolism, leading to increased systemic exposure of vonoprazan (Yamamoto et al., 2004), as well as reduces the risk of toxicity by inhibiting the formation of metabolites. Additionally, CYP3A4 inhibition may activate alternative metabolic pathways, leading to increased levels of the lesser toxic metabolite M-IV-Sul. Vonoprazan is primarily metabolized by CYP3A4 and to a lesser extent by CYP2B6, CYP2C19, CYP2D6, and SULT2A1 (Yamasaki et al., 2017). The vonoprazan metabolites M-I and M-III are formed via CYP3A4, and M-II is derived from M-I through non-oxidative pathways (Kogame et al., 2017), which exhibits higher toxicity than that of the parent compound. In contrast, M-IV-Sul is formed through SULT2A1 sulfate conjugation and CYP2C9 metabolism and less toxic than that of vonoprazan (Yamasaki et al., 2017; Echizen, 2016; Wang et al., 2022). Previous study showed that Clarithromycin exposure also increased when co-administered with vonoprazan, possibly due to synergistic PK interactions, without significant safety concerns (Sakurai et al., 2016). Tegoprazan, like vonoprazan, is primarily metabolized by CYP3A4 into its major active metabolite M1 (approximately 1/10 the activity of tegoprazan) (Han et al., 2021; Hwang et al., 2019). When co-administered with clarithromycin, AUC and Cmax increases not only of tegoprazan but also of M1; the possible explanation about the increase in AUC and Cmax of M1 is that clarithromycin is a potent inhibitor of CYP3A4 or P-gp may be involved in M1 metabolism (Gurley et al., 2008). Tegoprazan also significantly impacts 14-OH-clarithromycin absorption, prolonging Tmax and increasing AUC and Cmax but no notable effect on its t1/2. Since 14-OH-clarithromycin is metabolized by CYP3A4, competition with tegoprazan or its metabolite (M1) as a CYP3A4 substrate may increase the exposure of 14-OH-clarithromycin. Given that 14-OH-clarithromycin has greater antibacterial activity than that of clarithromycin, tegoprazan may improve antibacterial efficacy (Davey, 1991). P-CAB + clarithromycin is a strong choice for first-line therapy, even in borderline clarithromycin resistance scenarios, due to this synergy.

No PK changes were observed with both vonoprazan and tegoprazan when combined with amoxicillin likely because amoxicillin inhibits CYP2C8 (Niwa et al., 2016). Regarding the interaction with bismuth, vonoprazan showed no significant difference compared to lansoprazole or esomeprazole. When studied alone, tegoprazan significantly increased the AUC and Cmax of bismuth, which might be because of increased bismuth bioavailability due to decreased intragastric acidity by tegoprazan. There are few studies on the interaction of metronidazole, tetracycline, levofloxacin or rifabutin as antibiotics used for *H. pylori* infection with P-CABs, so further studies are needed. Jeon 2021 demonstrated a 37% decrease in tetracycline exposure with tegoprazan, likely due to high gastric pH hindering tetracycline’s absorption. This is a pharmacodynamic/absorption interaction, not metabolism-based. While data with PPIs are limited, it’s plausible that any potent acid suppression might lower tetracycline levels. Clinically, despite this reduction, P-CAB quadruple therapy remains effective, but it suggests that if a patient has malabsorption issues, or perhaps if using a less potent tetracycline dose, one might consider monitoring. In general, no dosage change is officially recommended; however, ensuring proper timing (avoid dairy, antacids) and full 14-day duration with tetracycline quadruple therapy is wise to counteract any absorption drop. Considering the PK interactions between P-CABs and antibiotics described above and patient-specific susceptibility tests, P-CAB-based treatment with appropriate therapeutic doses and duration would be helpful for eradication of *H. pylori*.

Most studies are conducted on vonoprazan regarding drug interactions related to the cytochrome enzymes inhibition. Vonoprazan inhibits the CYP2C19 metabolism and reduces cycloguanil (an active metabolite of proguanil) exposure, whereas tegoprazan does not significantly affect proguanil metabolism (Yang E. et al., 2023). The cycloguanil to proguanil ratio was lower in Koreans (0.35) than in Japanese population (0.84) (Funakoshi et al., 2019; Yang E. et al., 2023), possibly due to higher OCT1 deficiency alleles in Koreans, limiting hepatic uptake of proguanil and slowing its metabolic conversion to cycloguanil (Kang et al., 2007). Therefore, Koreans are recommended to take tegoprazan, having less CYP2C19 inhibitory effect, rather than vonoprazan when co-administering with proguanil (Ward et al., 1991). Since vonoprazan inhibits CYP3A, the blood concentration of tacrolimus is expected to be higher when co-administered with vonoprazan than with Rrabeprazole. However, two related studies showed contradictory results, and one study showed a similar levels of tacrolimus blood concentration in the CYP3A5*3/*3 group with reduced metabolic capacity of the CYP3A5 enzyme and more sensitivity to the inhibitory effect of vonoprazan. Care should be taken when vonoprazan administered in combination with CYP3A4 substrates, such as atorvastatin and midazolam, because this may lead to increased exposure of these drugs. Tegoprazan does not appear to have much interaction with CYP3A4 or 2C19 substrates; however, the lack of research on various drugs necessitates further research.

Considering that the PD of PCAB correlated well with AUC, there was no clinically significant food effect in the PK profile of vonoprazan, tegoprazan, and fexuprazan; however, food might have positive effect on keverprazan and zastaprazan, increasing total drug exposure. This suggests a positive food effect on its absorption, possibly due to enhanced dissolution or reduced first-pass metabolism in fed state.

P-CAB drugs are mainly developed and marketed in Asia; therefore, it is important to maintain the same effect in other ethnic groups. Ethnic variations in drug responses result from both internal factors, including genetic differences (polymorphism of metabolic enzymes or transporters) and body weight variations, as well as external factors, including environmental influences such as diet, lifestyle, and climate (Yasuda et al., 2008; Ling and Lee, 2011; International Conference on Harmonisation, 1998). However, Studies have demonstrated that vonoprazan maintains similar PK profile in Japanese and Caucasian populations (Jenkins et al., 2015; Sakurai et al., 2015). Similarly, fexuprazan exhibits consistent PK profile in Korean, Japanese, and Caucasian populations (Hwang et al., 2020). Various formulations have been developed to increase the stability or convenience of taking medication, and P-CAB exhibits the advantage of being resistant to degradation by stomach acid, eliminating the need for enteric coating and allowing for rapid onset of efficacy. Due to these characteristics, it is considered feasible to administer P-CAB in the form of orally disintegrating tablets, divided tablets, or powders. In particular, extensive safety studies have been conducted on various formulations of tegoprazan. These studies have confirmed that using a 1:1 ratio of delayed release to immediate-release not only facilitates an immediate increase in pH following administration but also provides continuous control of nighttime heartburn.

Our research has several limitations: First, the number of studies included in the analysis was small, the sample size of each study was small, and the follow-up period was limited. Therefore, evaluation of safety-related long-term PD and PK drug interaction is needed in many patients in the future. Second, the variable Tmax was modified during the study, leading to finding of changes absent in the original research. Consequently, the analysis primarily focused on Cmax and AUC. Third, despite applying a random-effects model, significant heterogeneity was observed in the results. Forth, due to the small sample size, quality assessment of the included studies was not performed, and Funnel plot and Egger’s test were not conducted to assess publication bias. Nonetheless, this review combines the PK data of all currently available P-CABs, and a meta-analysis was performed on data from more than two studies.

The interaction of PPIs with other medications (such as antiplatelet agents, anticoagulants, and anticancer drugs) has been extensively investigated (Ben et al., 2022). In particular, the increasing use of non-vitamin K antagonists oral anticoagulants, metabolized by CYP3A4, requires further studies on the effect of P-CABs is needed. Future studies should investigate the potential drug interactions of P-CABs, identify the most suitable P-CABs for various conditions or concomitant medications, and compare the PK of different P-CABs through network meta-analyses to assess their efficacy and safety profiles. Moreover, further PK studies that directly compare standard-dose P-CABs to double-dose PPIs would further increase access to P-CABs use due to cost. PK studies of P-CABs have mostly been conducted in healthy individuals except for *H. pylori* eradication treatment, and only vonoprazan had shown drug interactions with osimertinib in patients with NSCLC and with tacrolimus in renal transplant recipient. Further studies on the effect of the patient’s pathological condition on the PK of P-CABs are also needed.

## 5 Conclusion

This review demonstrated that special caution is warranted when P-CABs are administered concurrently with CYP3A4 inhibitors, and vonoprazan has been identified to have the potential to inhibit the CYP3A and CYP2C19 enzymes. Moreover, P-CABs exhibited minimal interaction with NSAIDs or aspirin and were largely unaffected by food intake, with the exceptions of keverprazan and zastaprazan.

## Data Availability

The original contributions presented in the study are included in the article/Supplementary Material, further inquiries can be directed to the corresponding author.

## References

[B1] Antequera C. M. Orleck K. Jacob R. Kenneally A. Wright W. L. (2024). Potassium-competitive acid blockers: rethinking acid suppression for gastroesophageal reflux disease and *Helicobacter pylori* . Postgrad. Med. 136 (2), 131–140. 10.1080/00325481.2024.2320081 38385191

[B2] Ben G. I. Luu M. Bardou M. (2022). An update on drug-drug interactions associated with proton pump inhibitors. Expert Opin. Drug Metab. Toxicol. 18 (5), 337–346. 10.1080/17425255.2022.2098107 35787720

[B3] Choi H. Y. Noh Y. H. Jin S. J. Kim Y. H. Kim M. J. Sung H. (2012). Bioavailability and tolerability of combination treatment with revaprazan 200 mg + itopride 150 mg: a randomized crossover study in healthy male Korean volunteers. Clin. Ther. 34 (9), 1999–2010. 10.1016/j.clinthera.2012.07.004 22858177

[B4] Cicala M. Emerenziani S. Guarino M. P. Ribolsi M. (2013). Proton pump inhibitor resistance, the real challenge in gastro-esophageal reflux disease. World J. Gastroenterol. 19 (39), 6529–6535. 10.3748/wjg.v19.i39.6529 24151377 PMC3801364

[B5] Daly A. K. (2015). Pharmacogenetics of drug metabolizing enzymes in the United Kingdom population: review of current knowledge and comparison with selected European populations. Drug Metabolism Personalized Ther. 30 (3), 165–174. 10.1515/dmdi-2014-0034 25803091

[B6] Davey P. G. (1991). The pharmacokinetics of clarithromycin and its 14-OH metabolite. J. Hosp. Infect. 19 (Suppl. A), 29–37. 10.1016/0195-6701(91)90215-t 1684980

[B7] Du Y. Yu L. Deng B. Li Q. Hu J. Li L. (2024). Pharmacokinetic interactions between tegoprazan and the combination of clarithromycin, amoxicillin and bismuth in healthy Chinese subjects: an open-label, single-center, multiple-dosage, self-controlled, phase I trial. Clin. Drug Investig. 44 (5), 343–355. 10.1007/s40261-024-01359-x 38615091

[B8] Echizen H. (2016). The first-in-class potassium-competitive acid blocker, vonoprazan fumarate: pharmacokinetic and pharmacodynamic considerations. Clin. Pharmacokinet. 55 (4), 409–418. 10.1007/s40262-015-0326-7 26369775

[B9] Eusebi L. H. Ratnakumaran R. Yuan Y. Solaymani-Dodaran M. Bazzoli F. Ford A. C. (2018). Global prevalence of, and risk factors for, gastro-oesophageal reflux symptoms: a meta-analysis. Gut 67 (3), 430–440. 10.1136/gutjnl-2016-313589 28232473

[B68] Fang Y. Lou D. Zhou J. Zhang Q. Dai Y. Ren W. (2024). Efficacy and safety of potassium-competitive acid blockers versus proton pump inhibitors in treating erosive esophagitis: a meta-analysis based on randomized controlled trials. J. Clin. Gastroenterol. 58 (9), 841–850. 10.1097/MCG.0000000000002052 39083496

[B11] Funakoshi R. Tomoda Y. Kudo T. Furihata K. Kusuhara H. Ito K. (2019). Effects of proton pump inhibitors, esomeprazole and vonoprazan, on the disposition of proguanil, a CYP2C19 substrate, in healthy volunteers. Br. J. Clin. Pharmacol. 85 (7), 1454–1463. 10.1111/bcp.13914 30845361 PMC6595331

[B12] Ganoci L. Božina T. Mirošević Skvrce N. Lovrić M. Mas P. Božina N. (2017). Genetic polymorphisms of cytochrome P450 enzymes: CYP2C9, CYP2C19, CYP2D6, CYP3A4, and CYP3A5 in the Croatian population. Drug Metab. Pers. Ther. 32 (1), 11–21. 10.1515/dmpt-2016-0024 28272018

[B13] Ghim J. L. Chin M. C. Jung J. Lee J. Kim S. Kim B. (2021). Pharmacokinetics and pharmacodynamics of tegoprazan coadministered with amoxicillin and clarithromycin in healthy subjects. J. Clin. Pharmacol. 61 (7), 913–922. 10.1002/jcph.1805 33341955

[B14] Gurley B. J. Swain A. Williams D. K. Barone G. Battu S. K. (2008). Gauging the clinical significance of P-glycoprotein-mediated herb-drug interactions: comparative effects of St. John's wort, Echinacea, clarithromycin, and rifampin on digoxin pharmacokinetics. Mol. Nutr. Food Res. 52 (7), 772–779. 10.1002/mnfr.200700081 18214850 PMC2562898

[B15] Han S. Choi H. Y. Kim Y. H. Nam J. Y. Kim B. Song G. S. (2021). Effect of food on the pharmacokinetics and pharmacodynamics of a single oral dose of tegoprazan. Clin. Ther. 43 (8), 1371–1380. 10.1016/j.clinthera.2021.06.007 34246485

[B16] Hozo S. P. Djulbegovic B. Hozo I. (2005). Estimating the mean and variance from the median, range, and the size of a sample. BMC Med. Res. Methodol. 5, 13. 10.1186/1471-2288-5-13 15840177 PMC1097734

[B17] Huh K. Y. Chung H. Kim Y. K. Lee S. Bhatia S. Takanami Y. (2022). Evaluation of safety and pharmacokinetics of bismuth-containing quadruple therapy with either vonoprazan or lansoprazole for *Helicobacter pylori* eradication. Br. J. Clin. Pharmacol. 88 (1), 138–144. 10.1111/bcp.14934 34080718 PMC9291775

[B18] Hwang I. Ji S. C. Oh J. Kim H. Cha H. Kim J. (2023). Randomised clinical trial: safety, tolerability, pharmacodynamics and pharmacokinetics of zastaprazan (JP-1366), a novel potassium-competitive acid blocker, in healthy subjects. Aliment. Pharmacol. Ther. 57 (7), 763–772. 10.1111/apt.17406 36732884

[B19] Hwang J. G. Jeon I. Park S. A. Lee A. Yu K. S. Jang I. J. (2020). Pharmacodynamics and pharmacokinetics of DWP14012 (fexuprazan) in healthy subjects with different ethnicities. Alimentary Pharmacol. Ther. 52 (11-12), 1648–1657. 10.1111/apt.16131 33111337

[B20] Hwang J. G. Yoo H. Lee J. W. Song G. S. Lee S. Kim M. G. (2019). Comparison of pharmacokinetic characteristics of two tegoprazan (CJ-12420) formulations in healthy male subjects. Transl. Clin. Pharmacol. 27 (2), 80–85. 10.12793/tcp.2019.27.2.80 32055586 PMC6989242

[B21] Hwang S. Ko J. W. Lee H. Kim S. Kim B. Song G. S. (2021). Co-administration of vonoprazan, not tegoprazan, affects the pharmacokinetics of atorvastatin in healthy male subjects. Front. Pharmacol. 12, 754849. 10.3389/fphar.2021.754849 34867368 PMC8632694

[B22] International Conference on Harmonisation (1998). Ethnic Factors in the Acceptability of Foreign Clinical Data (ICH E5). Available from: https://database.ich.org/sites/default/files/E5_R1__Guideline.pdf 10180134

[B23] Jenkins H. Jenkins R. Patat A. (2017). Effect of multiple oral doses of the potent CYP3A4 inhibitor clarithromycin on the pharmacokinetics of a single oral dose of vonoprazan: a phase I, open-label, sequential design study. Clin. Drug Investig. 37 (3), 311–316. 10.1007/s40261-016-0488-6 27928738

[B24] Jenkins H. Sakurai Y. Nishimura A. Okamoto H. Hibberd M. Jenkins R. (2015). Randomised clinical trial: safety, tolerability, pharmacokinetics and pharmacodynamics of repeated doses of TAK-438 (vonoprazan), a novel potassium-competitive acid blocker, in healthy male subjects. Aliment. Pharmacol. Ther. 41 (7), 636–648. 10.1111/apt.13121 25707624 PMC4654261

[B25] Jeon J. Y. Kim S. Y. Moon S. J. Oh K. Lee J. Kim B. (2021). Pharmacokinetic interactions between tegoprazan and metronidazole/tetracycline/bismuth and safety assessment in healthy Korean male subjects. Clin. Ther. 43 (4), 722–734. 10.1016/j.clinthera.2021.01.026 33637332

[B26] Kang H. J. Song I. S. Shin H. J. Kim W. Y. Lee C. H. Shim J. C. (2007). Identification and functional characterization of genetic variants of human organic cation transporters in a Korean population. Drug Metab. Dispos. 35 (4), 667–675. 10.1124/dmd.106.013581 17220237

[B27] Kanu J. E. Soldera J. (2024). Treatment of *Helicobacter pylori* with potassium competitive acid blockers: a systematic review and meta-analysis. World J. Gastroenterol. 30 (9), 1213–1223. 10.3748/wjg.v30.i9.1213 38577188 PMC10989498

[B28] Katz P. O. Dunbar K. B. Schnoll-Sussman F. H. Greer K. B. Yadlapati R. Spechler S. J. (2022). ACG clinical guideline for the diagnosis and management of gastroesophageal reflux disease. Am. J. Gastroenterol. 117 (1), 27–56. 10.14309/ajg.0000000000001538 34807007 PMC8754510

[B29] Katz P. O. Scheiman J. M. Barkun A. N. (2006). Review article: acid-related disease--what are the unmet clinical needs? Aliment. Pharmacol. Ther. 23 (Suppl. 2), 9–22. 10.1111/j.1365-2036.2006.02944.x 16700899

[B30] Kim H. S. Choi Y. K. Oh M. Cho Y. S. Ghim J. L. (2024). Enhancing drug administration flexibility: evaluation of pharmacokinetic properties of tegoprazan orally disintegrating tablet (ODT) administered via nasogastric tube or oral dosing. Transl. Clin. Pharmacol. 32 (2), 98–106. 10.12793/tcp.2024.32.e9 38974342 PMC11224899

[B31] Kogame A. Takeuchi T. Nonaka M. Yamasaki H. Kawaguchi N. Bernards A. (2017). Disposition and metabolism of TAK-438 (vonoprazan fumarate), a novel potassium-competitive acid blocker, in rats and dogs. Xenobiotica 47 (3), 255–266. 10.1080/00498254.2016.1182667 27225050

[B32] Lee J. A. Goak I. S. Lee J. Kim B. Moon S. J. Kwak Y. G. (2023). Evaluation of the comparative pharmacokinetic properties of a new orally disintegrating tablet of tegoprazan in healthy Korean subjects. Int. J. Clin. Pharmacol. Ther. 61 (9), 410–420. 10.5414/CP204378 37382330

[B33] Ling W. H. Lee S. C. (2011). Inter-ethnic differences--how important is it in cancer treatment? Ann. Acad. Med. Singap 40 (8), 356–361. 10.47102/annals-acadmedsg.v40n8p356 22065001

[B34] Marshall B. J. Warren J. R. (1984). Unidentified curved bacilli in the stomach of patients with gastritis and peptic ulceration. Lancet 1 (8390), 1311–1315. 10.1016/s0140-6736(84)91816-6 6145023

[B35] Mei T. Noguchi H. Suetsugu K. Hisadome Y. Kaku K. Okabe Y. (2020). Effects of concomitant administration of vonoprazan fumarate on the tacrolimus blood concentration in kidney transplant recipients. Biol. Pharm. Bull. 43 (10), 1600–1603. 10.1248/bpb.b20-00361 32999170

[B36] Miao J. Hu C. Tang J. Wang W. Wang Y. Men R. (2023). Pharmacokinetics, safety, and tolerability of vonoprazan- or esomeprazole-based bismuth-containing quadruple therapy: a phase 1, double-blind, parallel-group study in adults with *Helicobacter pylori* infection in China. Clin. Pharmacol. Drug Dev. 12 (10), 1036–1044. 10.1002/cpdd.1276 37443412

[B37] Moon S. J. Shin N. Kang M. Kim B. Kim M. G. (2022). Pharmacokinetic interactions between tegoprazan and naproxen, aceclofenac, and celecoxib in healthy Korean male subjects. Clin. Ther. 44 (7), 930–44.e1. 10.1016/j.clinthera.2022.06.002 35787943

[B38] Mulford D. J. Leifke E. Hibberd M. Howden C. W. (2022). The effect of food on the pharmacokinetics of the potassium-competitive acid blocker vonoprazan. Clin. Pharmacol. Drug Dev. 11 (2), 278–284. 10.1002/cpdd.1009 34431240 PMC9291755

[B39] Mulford D. J. Ramsden D. Zhang L. Michon I. Leifke E. Smith N. (2023). Tiered approach to evaluate the CYP3A victim and perpetrator drug–drug interaction potential for vonoprazan using PBPK modeling and clinical data to inform labeling. CPT Pharmacometrics Syst. Pharmacol. 12 (4), 532–544. 10.1002/psp4.12939 36896795 PMC10088082

[B40] Niwa T. Morimoto M. Hirai T. Hata T. Hayashi M. Imagawa Y. (2016). Effect of penicillin-based antibiotics, amoxicillin, ampicillin, and piperacillin, on drug-metabolizing activities of human hepatic cytochromes P450. J. Toxicol. Sci. 41 (1), 143–146. 10.2131/jts.41.143 26763401

[B41] Oh J. Yang E. Jang I. J. Lee H. Yoo H. Chung J. Y. (2023). Pharmacodynamic and pharmacokinetic drug interactions between fexuprazan, a novel potassium-competitive inhibitor, and aspirin, in healthy subjects. Pharmaceutics 15 (2), 549. 10.3390/pharmaceutics15020549 36839870 PMC9958674

[B42] Oh M. Lee H. Kim S. Kim B. Song G. S. Shin J. G. (2023). Evaluation of pharmacokinetic drug-drug interaction between tegoprazan and clarithromycin in healthy subjects. Transl. Clin. Pharmacol. 31 (2), 114–123. 10.12793/tcp.2023.31.e11 37440779 PMC10333645

[B43] Park S. Yang E. Kim B. Kwon J. Jang I. J. Lee S. H. (2023). Pharmacokinetic and pharmacodynamic exploration of various combinations of tegoprazan immediate and delayed-release formulations. Br. J. Clin. Pharmacol. 89 (9), 2877–2887. 10.1111/bcp.15784 37170677

[B44] Patel A. Laine L. Moayyedi P. Wu J. (2024). AGA clinical practice update on integrating potassium-competitive acid blockers into clinical practice: expert review. Gastroenterology 167 (6), 1228–1238. 10.1053/j.gastro.2024.06.038 39269391

[B45] Sakurai Y. Nishimura A. Kennedy G. Hibberd M. Jenkins R. Okamoto H. (2015). Safety, tolerability, pharmacokinetics, and pharmacodynamics of single rising TAK-438 (vonoprazan) doses in healthy male Japanese/non-Japanese subjects. Clin. Transl. Gastroenterol. 6 (6), e94. 10.1038/ctg.2015.18 26111126 PMC4816246

[B46] Sakurai Y. Shiino M. Horii S. Okamoto H. Nakamura K. Nishimura A. (2017). Pharmacokinetic drug–drug interactions between vonoprazan and low-dose aspirin or nonsteroidal anti-inflammatory drugs: a phase 2, open-label, study in healthy Japanese men. Clin. Drug Investig. 37 (1), 39–49. 10.1007/s40261-016-0455-2 PMC5209422 27581248

[B47] Sakurai Y. Shiino M. Okamoto H. Nishimura A. Nakamura K. Hasegawa S. (2016). Pharmacokinetics and safety of triple therapy with vonoprazan, amoxicillin, and clarithromycin or metronidazole: a phase 1, open-label, randomized, crossover study. Adv. Ther. 33 (9), 1519–1535. 10.1007/s12325-016-0374-x 27432383

[B48] Seo S. Jung H. K. Gyawali C. P. Lee H. A. Lim H. S. Jeong E. S. (2024). Treatment response with potassium-competitive acid blockers based on clinical phenotypes of gastroesophageal reflux disease: a systematic literature review and meta-analysis. J. Neurogastroenterol. Motil. 30 (3), 259–271. 10.5056/jnm24024 38972863 PMC11238110

[B49] Shin J. M. Kim N. (2013). Pharmacokinetics and pharmacodynamics of the proton pump inhibitors. J. Neurogastroenterol. Motil. 19 (1), 25–35. 10.5056/jnm.2013.19.1.25 23350044 PMC3548122

[B50] Shin W. Yang A. Y. Park H. Lee H. Yoo H. Kim A. (2023). A comparative pharmacokinetic study of fexuprazan 10 mg: demonstrating bioequivalence with the reference formulation and evaluating steady state. Pharmaceuticals 16 (8), 1141. 10.3390/ph16081141 37631056 PMC10458111

[B51] Sunwoo J. Oh J. Moon S. J. Ji S. C. Lee S. H. Yu K. S. (2018). Safety, tolerability, pharmacodynamics and pharmacokinetics of DWP14012, a novel potassium-competitive acid blocker, in healthy male subjects. Alimentary Pharmacol. Ther. 48 (2), 206–218. 10.1111/apt.14818 29863280

[B52] Vakil N. van Zanten S. V. Kahrilas P. Dent J. Jones R. Global Consensus Group (2006). The Montreal definition and classification of gastroesophageal reflux disease: a global evidence-based consensus. Am. J. Gastroenterol. 101 (8), 1900–1943. 10.1111/j.1572-0241.2006.00630.x 16928254

[B53] Wang M. S. Gong Y. Zhuo L. S. Shi X. X. Tian Y. G. Huang C. K. (2022). Distribution- and metabolism-based drug discovery: a potassium-competitive acid blocker as a proof of concept. Res. (Wash D C) 2022, 9852518. 10.34133/2022/9852518 PMC9343080 35958113

[B54] Wang W. X. Li R. J. Li X. F. (2024). Efficacy and safety of potassium-competitive acid blockers vs proton pump inhibitors for peptic ulcer disease or postprocedural artificial ulcers: a systematic review and meta-analysis. Clin. Transl. Gastroenterology 15, e1. 10.14309/ctg.0000000000000754 PMC11421725 39072507

[B55] Ward S. A. Helsby N. A. Skjelbo E. Brøsen K. Gram L. F. Breckenridge A. M. (1991). The activation of the biguanide antimalarial proguanil co-segregates with the mephenytoin oxidation polymorphism--a panel study. Br. J. Clin. Pharmacol. 31 (6), 689–692. 10.1111/j.1365-2125.1991.tb05594.x 1867963 PMC1368581

[B56] Watari S. Araki M. Matsumoto J. Yoshinaga K. Sekito T. Maruyama Y. (2021). Blood concentrations of tacrolimus upon conversion from rabeprazole to vonoprazan in renal transplant recipients: correlation with cytochrome P450 gene polymorphisms. Drug Metabolism Pharmacokinet. 40, 100407. 10.1016/j.dmpk.2021.100407 34352707

[B57] Won H. Kim E. Chae J. Lee H. Cho J. Y. Jang I. J. (2024). Pharmacokinetic interactions between fexuprazan, a potassium-competitive acid blocker, and nonsteroidal anti-inflammatory drugs in healthy males. Clin. Transl. Sci. 17 (5), e13798. 10.1111/cts.13798 38700290 PMC11067709

[B58] Yamade M. Suzuki T. Kagami T. Ichikawa H. Uotani T. Shirai N. (2017). Potassium-competitive acid blocker versus proton pump inhibitor as first-line triple therapy for helicobacter pylori eradication: a systematic review and meta-analysis. Gastroenterology 152 (5), S151–S152. Available from: https://www.embase.com/records?subaction=viewrecord&id=L618670573

[B59] Yamamoto F. Harada S. Mitsuyama T. Harada Y. Kitahara Y. Yoshida M. (2004). Concentration of clarithromycin and 14-R-hydroxy-clarithromycin in plasma of patients with *Mycobacterium avium* complex infection, before and after the addition of rifampicin. Jpn. J. Antibiot. 57 (1), 124–133. Available from: https://www.jstage.jst.go.jp/article/antibiotics1968b/57/1/57_124/_pdf 15116577

[B60] Yamasaki H. Kawaguchi N. Nonaka M. Takahashi J. Morohashi A. Hirabayashi H. (2017). *In vitro* metabolism of TAK-438, vonoprazan fumarate, a novel potassium-competitive acid blocker. Xenobiotica 47 (12), 1027–1034. 10.1080/00498254.2016.1203505 27414183

[B61] Yang A. Y. Yoo H. Shin W. Lee Y. S. Lee H. Kim S. E. (2023). Size-reduced fexuprazan 20 mg demonstrated the optimal bioavailability and bioequivalence with the reference formulation. Transl. Clin. Pharmacol. 31 (1), 40–48. 10.12793/tcp.2023.31.e3 37034124 PMC10079506

[B62] Yang E. Ji S. C. Jang I. J. Lee S. (2023). Evaluation of CYP2C19-mediated pharmacokinetic drug interaction of tegoprazan, compared with vonoprazan or esomeprazole. Clin. Pharmacokinet. 62 (4), 599–608. 10.1007/s40262-023-01228-4 36897544 PMC10085907

[B63] Yasuda S. U. Zhang L. Huang S. M. (2008). The role of ethnicity in variability in response to drugs: focus on clinical pharmacology studies. Clin. Pharmacol. Ther. 84 (3), 417–423. 10.1038/clpt.2008.141 18615002

[B64] Yokota H. Sato K. Sakamoto S. Okuda Y. Fukuda N. Asano M. (2022). Effects of CYP3A4/5 and ABC transporter polymorphisms on osimertinib plasma concentrations in Japanese patients with non-small cell lung cancer. Invest. New Drugs 40 (6), 1254–1262. 10.1007/s10637-022-01304-9 36149549

[B65] Yoon D. Y. Sunwoo J. Shin N. Kim A. R. Kim B. Song G. S. (2021). Effect of meal timing on pharmacokinetics and pharmacodynamics of tegoprazan in healthy male volunteers. Clin. Transl. Sci. 14 (3), 934–941. 10.1111/cts.12958 33382926 PMC8212751

[B66] Zhou S. Xie L. Zhou C. Zhao Y. Wang L. Ding S. (2023). Keverprazan, a novel potassium-competitive acid blocker: single ascending dose safety, tolerability, pharmacokinetics, pharmacodynamics and food effect in healthy subjects. Eur. J. Pharm. Sci. 190, 106578. 10.1016/j.ejps.2023.106578 37666458

[B67] Zou S. Ouyang M. Cheng Q. Shi X. Sun M. (2024). Acid-suppressive drugs: a systematic review and network meta-analysis of their nocturnal acid-inhibitory effect. Pharmacotherapy 44 (2), 171–183. 10.1002/phar.2899 38049205

