# Supplementary material for: Clinical pharmacokinetics of potassium competitive acid blockers: a systematic review and meta-analysis
Source: Front Pharmacol. 2025 Jul 8;16:1580969. doi: 10.3389/fphar.2025.1580969 (PMC12280725; doi:10.3389/fphar.2025.1580969)
Supplement: Supplementary file 1 [file Supplementaryfile1.docx]

**Clinical pharmacokinetics of potassium competitive acid blockers: a systematic review and meta-analysis**

Jiaqi Liu ^1^, Jongsung Hahn^1^

^1^Department of Pharmacy, Jeonbuk National University, Jeonju, Jeollabuk 54896, Republic of Korea

**Corresponding author:** Professor Jongsung Hahn,

Department of Pharmacy, Jeonbuk National University, 567 Baekje‑daero, Deokjin, Jeonju, Jeollabuk 54896, Republic of Korea

E‑mail: jongsung@jbnu.ac.kr

(a)


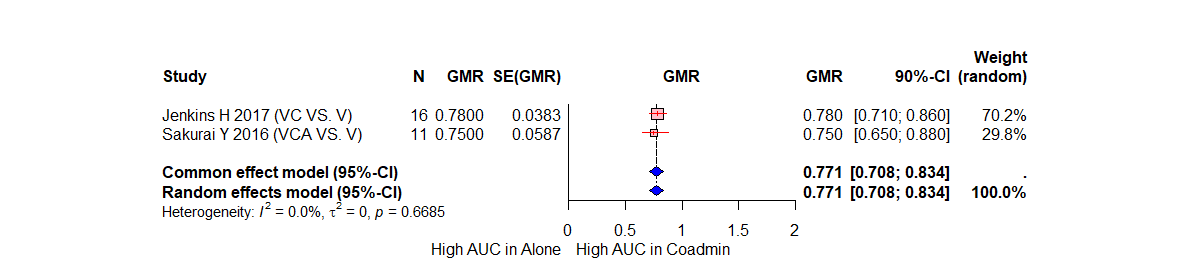


(b)


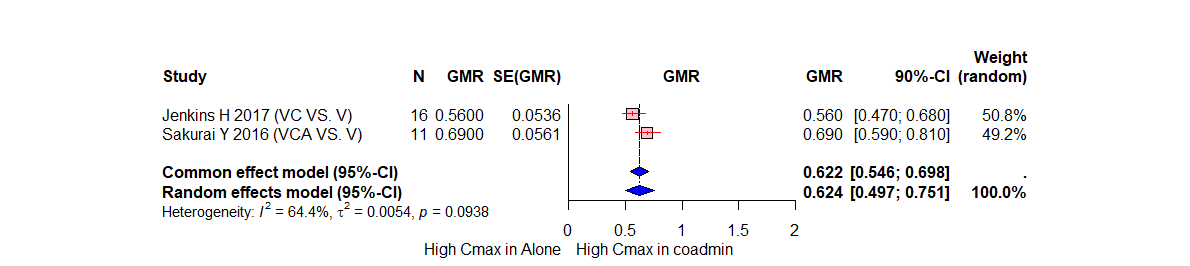


(c)


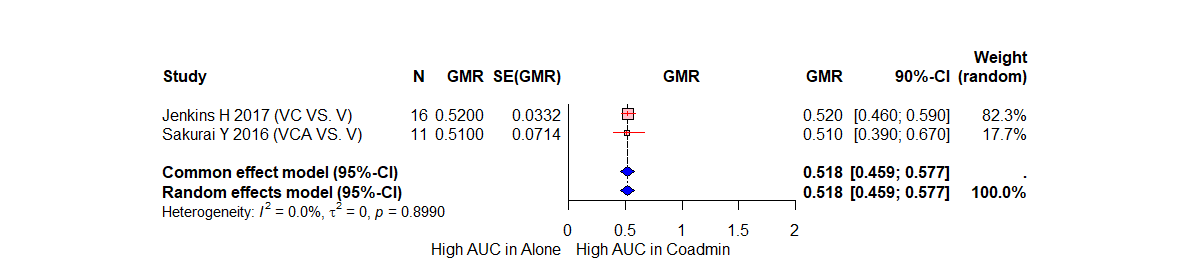


(d)


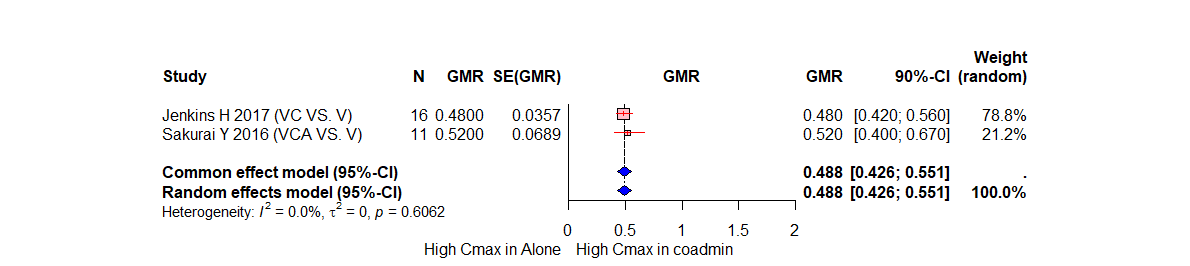


(e)


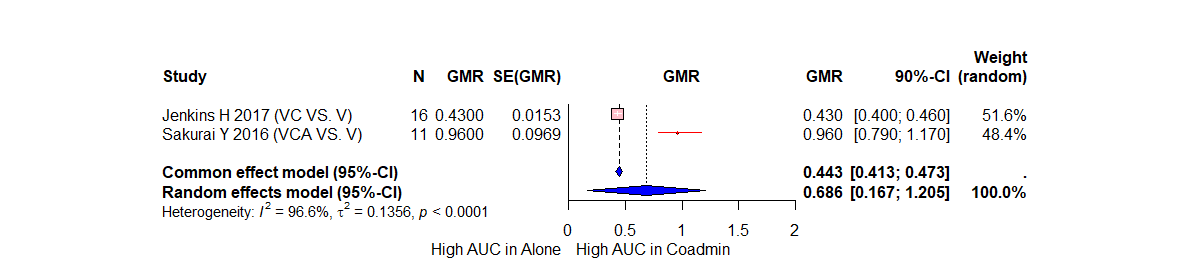


(f)


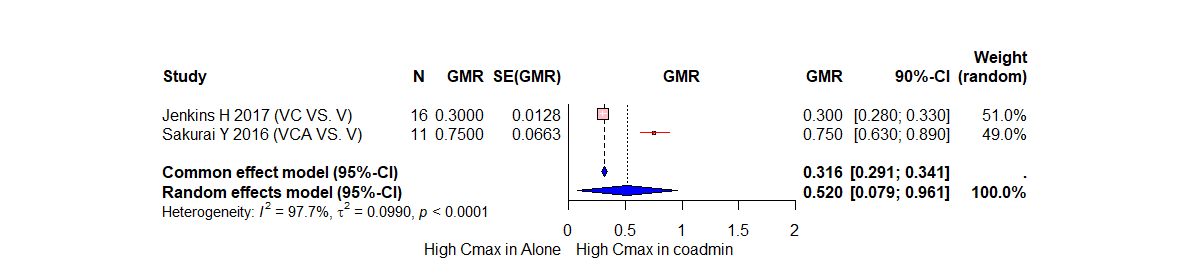


(g)


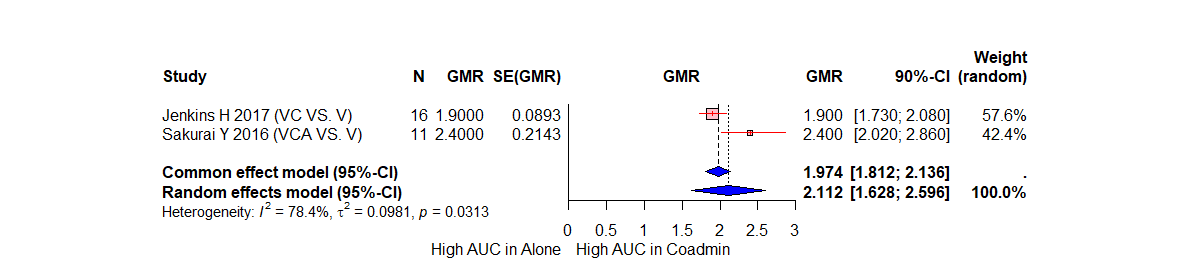


(h)


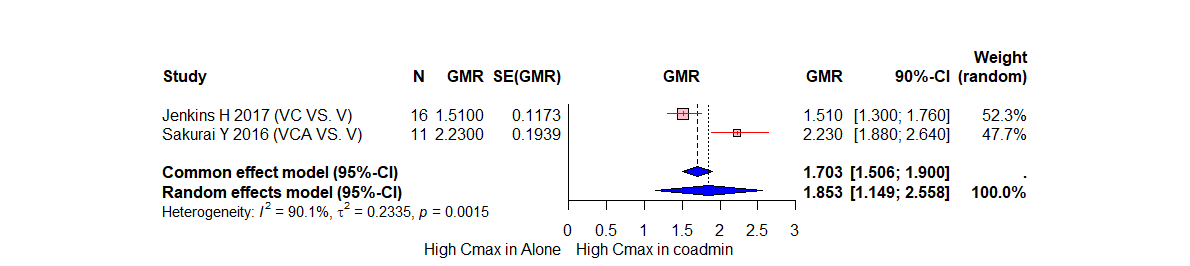


**Fig. S1** Forest plot showing the changes in PK parameters of vonoprazan metabolites when administered alone and co-administered with other drugs. **a** GMR of AUC of M-I. **b** GMR of Cmax of M-I. **c** GMR of AUC of M-II. **d** GMR of Cmax of M-II. **e** GMR of AUC of M-III. **f** GMR of Cmax of M-III. **g** GMR of AUC of M-IV-Sul. **h** The GMR of Cmax of M-IV-Sul. AUC, area under the concentration curve; Cmax, peak concentration; CI, confidence interval; GMR, geometric mean ratio; SE, standard error; Coadmin: coadministration; V, vonoprazan; VC, vonoprazan and clarithromycin; VCA, vonoprazan and clarithromycin and amoxicillin

(a)


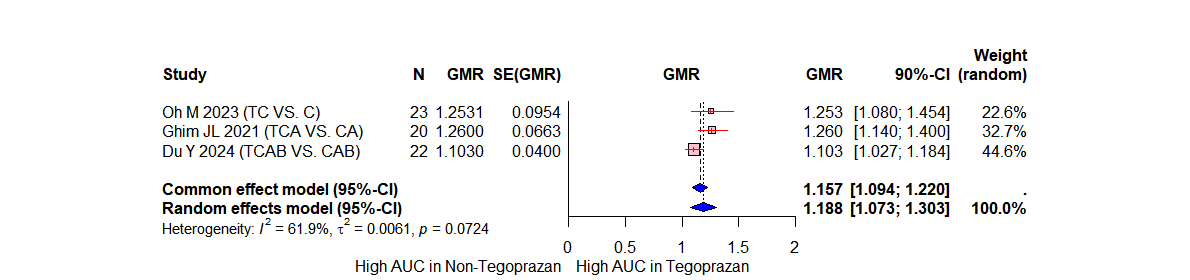


(b)


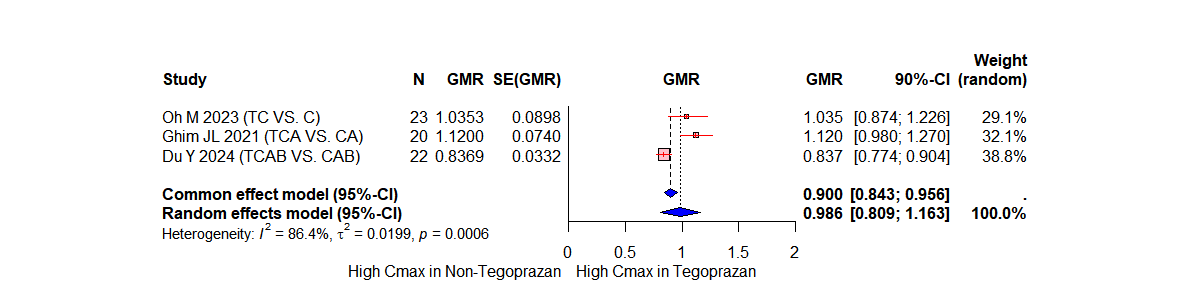


(c)


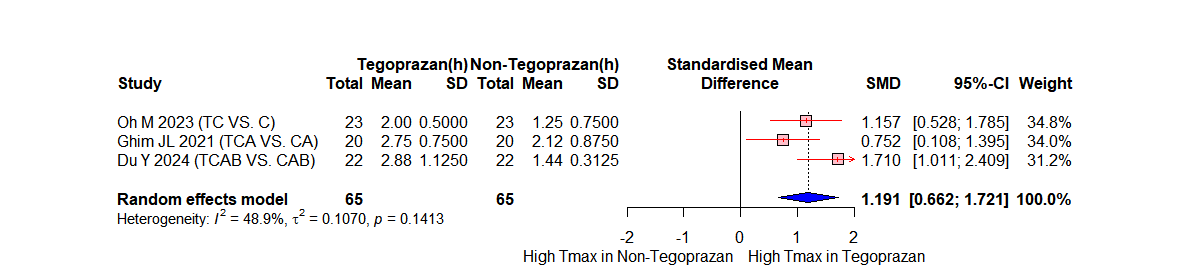


(d)


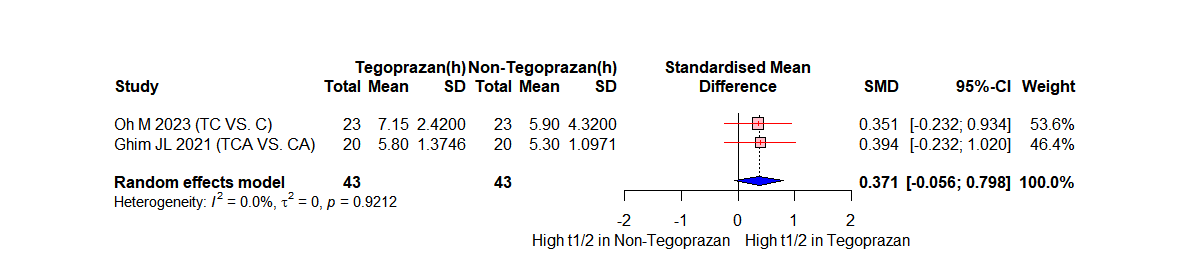


(e)


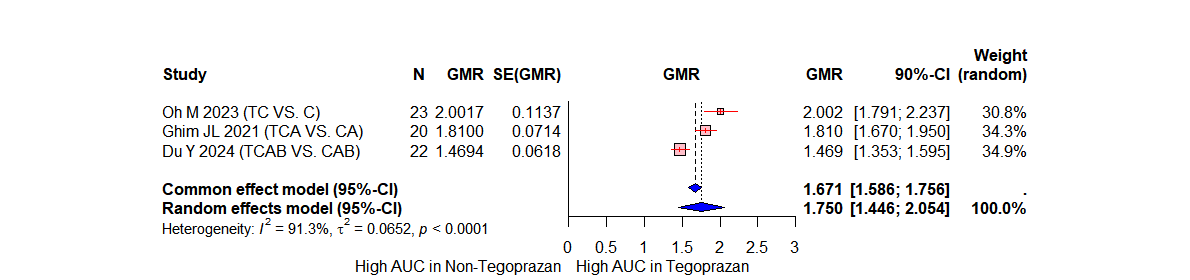


(f)


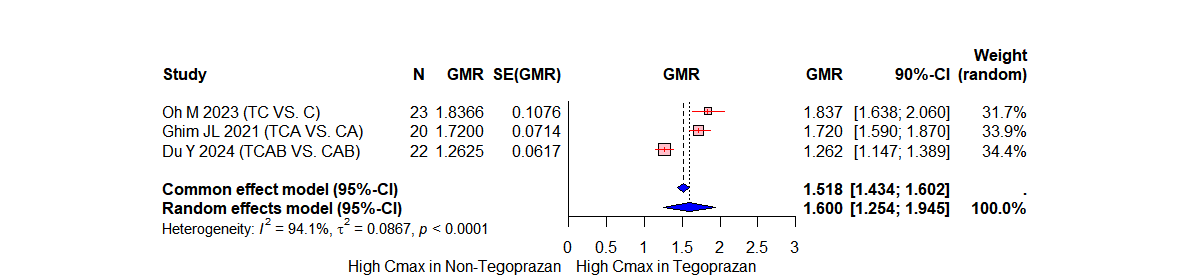


(g)


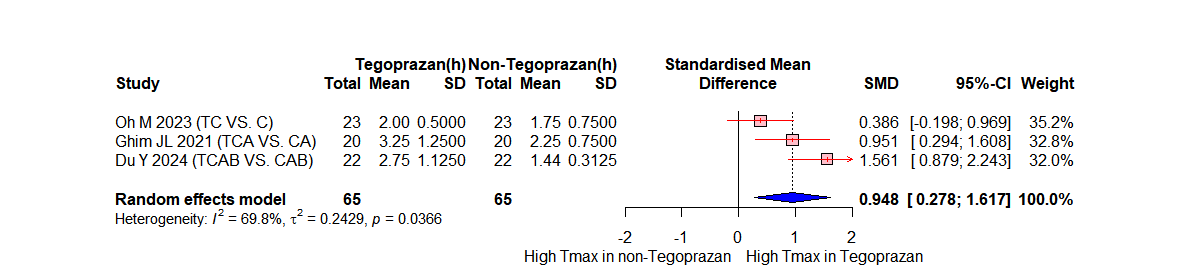


(h)


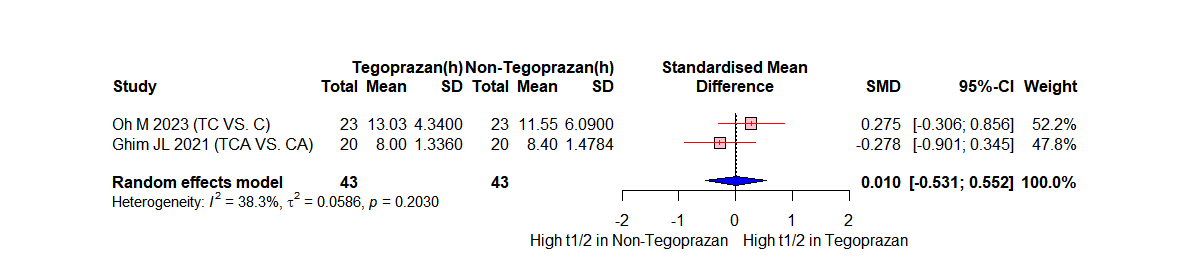


**Fig. S2** Forest plot showing changes in PK parameters of clarithromycin and 14-OH-clarithromycin when administered alone and co-administered with tegoprazan. **a** GMR of AUC of clarithromycin. **b** GMR of Cmax of clarithromycin. **c** SMD of Tmax of clarithromycin. **d** SMD of t1/2 of clarithromycin. **e** GMR of AUC of 14-OH-clarithromycin. **f** GMR of Cmax of 14-OH-clarithromycin. **g** SMD of Tmax of 14-OH-clarithromycin. **h** SMD of t1/2 of 14-OH-clarithromycin. AUC, area under the concentration curve; Cmax, peak concentration; t1/2, elimination half-­life; Tmax, time to reach Cmax; CI, confidence interval; GMR, geometric mean ratio; SD, standard deviation; SE, standard error; SMD, standard mean difference; TC, tegoprazan and clarithromycin; TCA, tegoprazan and clarithromycin and amoxicillin; TCAB, tegoprazan and clarithromycin and amoxicillin and bismuth; C, clarithromycin; CA, clarithromycin and amoxicillin; CAB, clarithromycin and amoxicillin and bismuth

(a)


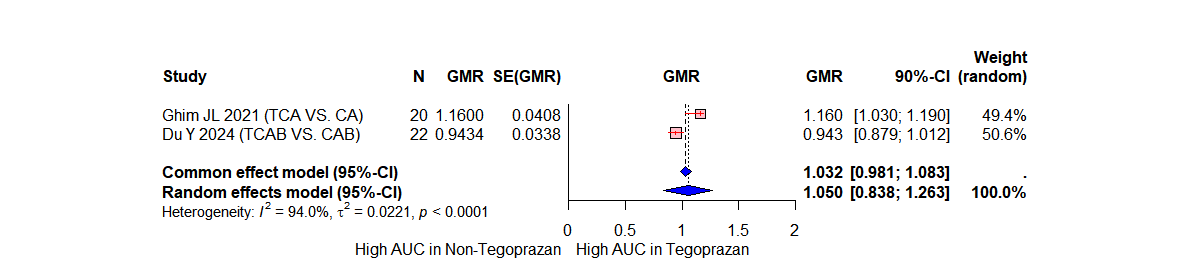


(b)


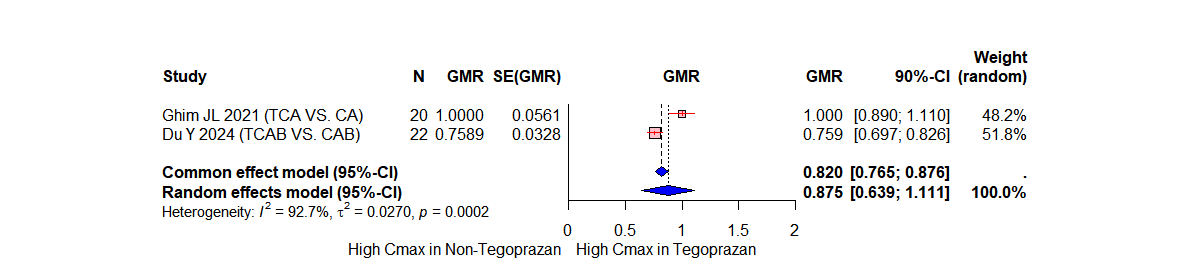


(c)


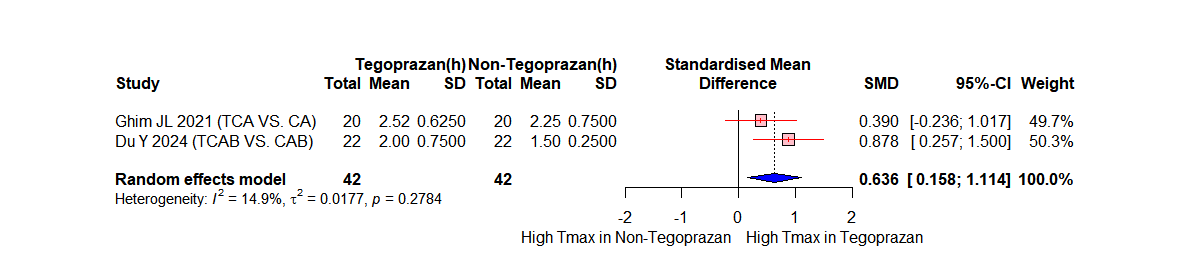


(d)


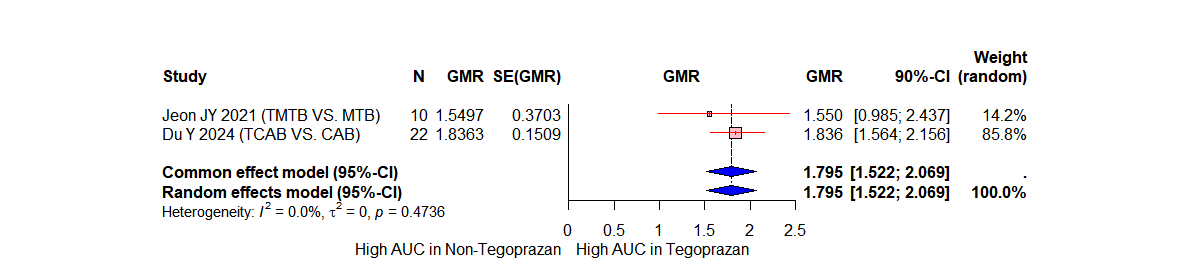


(e)


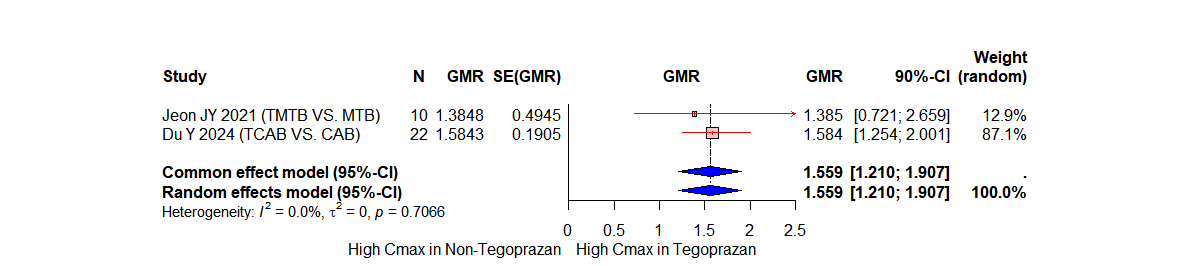


(f)


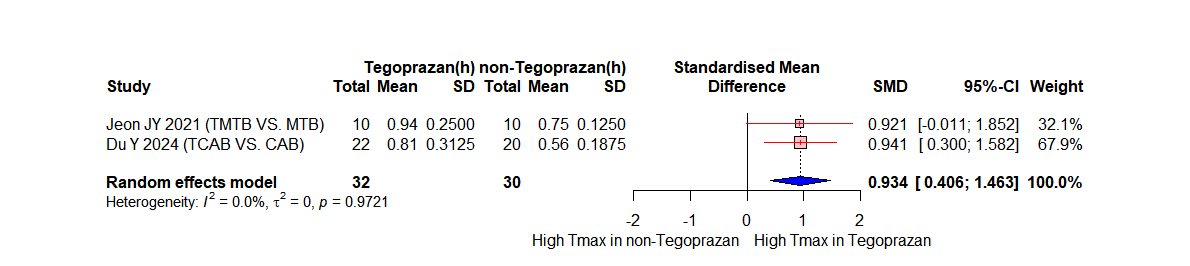


**Fig. S3** Forest plot showing changes in PK parameters of amoxicillin and bismuth when administered alone and co-administered with tegoprazan. **a** GMR of AUC of amoxicillin. **b** GMR of Cmax of amoxicillin. **c** SMD of Tmax of amoxicillin. **d** GMR of AUC of bismuth. **e** GMR of Cmax of bismuth. **f** SMD of Tmax of bismuth. AUC, area under the concentration curve; Cmax, peak concentration; Tmax, time to reach Cmax; CI, confidence interval; GMR, geometric mean ratio; SD, standard deviation; SE, standard error; SMD, standard mean difference; TCA, tegoprazan and clarithromycin and amoxicillin; TCAB, tegoprazan and clarithromycin and amoxicillin and bismuth; TMTB, tegoprazan and metronidazole and tetracycline and bismuth; CA, clarithromycin and amoxicillin; CAB, clarithromycin and amoxicillin and bismuth; MTB, tegoprazan and metronidazole and tetracycline

**Table S1** Characteristics of the included studies

| Study | | Patient Characteristics | | | Study Design | | | Outcome | |
| --- | --- | --- | --- | --- | --- | --- | --- | --- | --- |
| Author, year | Country | Sex | Age (mean), years | Comorbidity | Study design | Number of participants | Dosing regimen | Study style | Reported PK parameter |
| Revaprazan: | | | | | | | | | |
| Choi HY et al. 2012 | Korea | All male | 31.9 | Healthy | RCT | 30 | Revaprazan Vs. Revaprazan + Itopride  Itopride Vs. Revaprazan + Itopride | DDI | Cmax, Tmax, AUC, CL/F |
| Vonoprazan: | | | | | | | | | |
| Jenkins H et al. 2015 | Japan and UK | All male | 27 (Japanese) 28 (UK) | Healthy | RCT | 60 (Japan )  48 (UK) | Vonoprazan | Ethnicity | Cmax, Tmax, AUC, t1/2, CL/F, Rac |
| Sakurai Y et al. 2015 | Japan and UK | All male | 26 (Japanese) 26 (UK) | Healthy | RCT | 84 (Japan )  63 (UK ) | Vonoprazan | Ethnicity | Cmax, Tmax, AUC, t1/2, CL/F, Fe |
| Sakurai Y et al. 2016 | Japan | All male | 29.4 | Healthy | RCT | 12 | Vonoprazan Vs. Vonoprazan + Amoxicillin + Clarithromycin  Amoxicillin Vs. Vonoprazan + Amoxicillin + Clarithromycin  Clarithromycin VS.Vonoprazan + Amoxicillin + Clarithromycin | DDI | Cmax, Tmax, AUC, t1/2, CL/F, Vz/F, MRT |
|  | Japan | All male | 26.2 | Healthy | RCT | 12 | Vonoprazan Vs. Vonoprazan + Amoxicillin + Metronidazole  Amoxicillin Vs. Vonoprazan + Amoxicillin + Metronidazole  Metronidazole VS.Vonoprazan + Amoxicillin + Metronidazole | DDI | Cmax, Tmax, AUC, t1/2, CL/F, Vz/F, MRT |
| Jenkins H et al. 2017 | France | All male | 27.3 | Healthy | Non-RCT | 16 | Vonoprazan Vs. Vonoprazan + Clarithromycin  Clarithromycin Vs. Vonoprazan + Clarithromycin | DDI | Cmax, Tmax, AUC, t1/2, CL/F, Vz/F |
| Sakurai Y et al. 2017 | Japan | All male | 24.2 | Healthy | RCT | 8 | Vonoprazan Vs. Vonoprazan + Aspirin  Aspirin Vs. Vonoprazan + Aspirin  Vonoprazan Vs.Vonoprazan + Loxoprofen  Loxoprofen Vs. Vonoprazan + Loxoprofen  Vonoprazan Vs. Vonoprazan + Diclofenac  Diclofenac Vs. Vonoprazan + Diclofenac  Vonoprazan Vs. Vonoprazan + Meloxicam  Meloxicam Vs. Vonoprazan + Meloxicam | DDI | Cmax, Tmax, AUC, t1/2 |
| Funakoshi R et al. 2019 | Japan | All male | 37 (median) | Healthy | Non-RCT | 7 | Proguanil Vs. Vonoprazan + proguanil | DDI | Cmax, Tmax, AUC, t1/2, CL/F, CLr, Vz/F, MR |
| Mei T et al. 2020 | Japan | Male : Female = 35 : 17 | 55.7 | Kidney transplant recipients | Retrospective observational study | 52 | Rabeprazole + Tacrolimus Vs. Vonoprazan + Tacrolimus | DDI | Cmin |
| Hwang S et al. 2021 | Korea | All male | 33.9 | Healthy | RCT | 32 | Atorvastatin VS.Tegoprazan + Atorvastatin  Atorvastatin VS.Vonoprazan + Atorvastatin | DDI | Cmax, Tmax, AUC, MR |
| Watari S et al. 2021 | Japan | Male : Female = 34 : 18 | CYP3A5 genotype *1/*1 + *1/*3: 47 (median) CYP3A5 genotype *3/*3: 56 (median) | Kidney transplant recipients | Retrospective observational study | 52 | Rabeprazole + Tacrolimus Vs. Vonoprazan + Tacrolimus | DDI | Cmin |
| Huh KY et al. 2022 | Korea | Male : Female = 14 : 1 | Vonoprazan: 32.8 Lansoprazole: 33.3 | H.pylori positive patients | RCT | 30 | Vonoprazan + Bismuth + Clarithromycin + Amoxicillin Vs. Lansoprazole + Bismuth + Clarithromycin + Amoxicillin | DDI | Cmax, Tmax, AUC, t1/2, CL/F, CLr, Vz/F, fe |
| Laine L et al. 2022 | USA | Male : Female = 32 : 12 | 36 | Healthy | RCT | 40 | Vonoprazan Vs. Lansoprazole | Direct Comparison | Cmax, Tmax, AUC, t1/2 |
| Mulford DJ et al. 2022 | Northern Ireland | Male : Female = 12 : 12 | 25.5 | Healthy | RCT | 24 | Fed Vs. Fasted | Food effect | Cmax, Tmax, AUC, t1/2 |
| Yokota H et al. 2022 | Japan | Male : Female = 8 : 15 | 68.6 | Non-small cell lung cancer patients | Prospective observationalstudy | 23 | Osimertinib Vs. Vonoprazan+Osimertinib Vs. Esomeprazole+Osimertinib/Lafutidine+Osimertinib | DDI | Cmax, Tmax, AUC, Cmin |
| Miao J et al. 2023 | China | Male : Female = 27 : 17 | Vonoprazan: 34.5 Esomeprazole: 31.6 | H. pylori positive subjects | RCT | 44 | Vonoprazan + Bismuth + Clarithromycin + Amoxicillin Vs. Esomeprazole + Bismuth + Clarithromycin + Amoxicillin | DDI | Cmax, Tmax, AUC, t1/2, CL/F, CLr, fe, Aeτ |
| Mulford DJ et al. 2023 | America | Male : Female = 12 : 8 | 32.3 | Healthy | Non-RCT | 20 | Midazolam Vs. Midazolam+Vonoprazan | DDI | Cmax, Tmax, AUC, t1/2 |
| Yang E et al. 2023 | Korea | All male | 32.6 | Healthy | RCT | 19 | Proguanil Vs. Tegoprazan + Proguanil Vs. Esomeprazole + Proguanil Vs. Vonoprazan + Proguanil | DDI | Cmax, Tmax, AUC, t1/2, CL/F, fe, MR |
| Zhou S et al. 2023 | China | Male : Female = 12 : 14 | Keverprazan: 22.63 Vonoprazan: 26.00 | Healthy | RCT | 8 (Keverprazan)  4 (Vonoprazan) | Vonoprazan Vs. Keverprazan | Direct Comparison | Cmax, Tmax, AUC, t1/2, CL/F, Vz/F, MR, Tmin,  Cmin, CAVSS, Rac, DF |
| Tegoprazan: | | | | | | | | | |
| Hwang JG et al. 2019 | Korea | All male | 23.9 | Healthy | RCT | 12 | Two different 100 mg formulations | Formulation | Cmax, Tmax, AUC, t1/2, CL/F, Vz/F, MR |
| Ghim JL et al. 2021 | Korea | All male | 27.35 | Healthy | RCT | 24 | Tegoprazan Vs.. Tegoprazan + Amoxicillin + Clarithromycin  Amoxicillin + Clarithromycin Vs. Tegoprazan + Amoxicillin + Clarithromycin | DDI | Cmax, Tmax, AUC, t1/2, CL/F, Vz/F |
| Han S et al. 2021 | Korea | All male | 23.79 | Healthy | RCT | 24 | Fed Vs. Fasted | Food effect | Cmax, Tmax, AUC, t1/2 |
| Hwang S et al. 2021 | Korea | All male | 33.9 | Healthy | RCT | 32 | Atorvastatin VS.Tegoprazan + Atorvastatin VS.Vonoprazan + Atorvastatin | DDI | Cmax, Tmax, AUC, MR |
| Jeon JY et al. 2021 | Korea | All male | 23.94 | Healthy | RCT | 32 | Tegoprazan Vs. Tegoprazan + Metronidazole + Tetracycline + Bismuth  Metronidazole + Tetracycline + Bismuth Vs. Tegoprazan + Metronidazole + Tetracycline + Bismuth | DDI | Cmax, Tmax, AUC, t1/2, CL/F, Cmin, CAVSS |
| Yoon DY et al. 2021 | Korea | All male | 29.8 | Healthy | RCT | 12 | Fed Vs. Fasted | Food effect | Cmax, Tmax, AUC, t1/2, MR |
| Moon SJ et al. 2022 | Korea | All male | Tegoprazan - Naproxen: 23.92 Tegoprazan - Naproxen: 25.94 Tegoprazan - Celecoxib: 23.89 | Healthy | RCT | 60 | Tegoprazan Vs. Tegoprazan + Naproxen  Naproxen Vs. Tegoprazan + Naproxen Tegoprazan Vs. Tegoprazan + Aceclofenac  Aceclofenac Vs. Tegoprazan + Aceclofenac Tegoprazan Vs. Tegoprazan + Celecoxib  Celecoxib Vs. Tegoprazan + Celecoxib | DDI | Cmax, Tmax, AUC, t1/2, CL/F, Vz/F, Cmin, CAVSS |
| Lee JA et al. 2023 | Korea | Male : Female = 40 : 8 | 24.96 | Healthy | RCT | 48 | Immediate-release formulation Vs. orally disintegrating tablet | Formulation | Cmax, Tmax, AUC, t1/2, CL/F, Vz/F |
| Oh M et al. 2023 | Korea | All male | 24.79 | Healthy | RCT | 24 | Tegoprazan Vs. Tegoprazan + Clarithromycin  Clarithromycin Vs. Tegoprazan + Clarithromycin | DDI | Cmax, Tmax, AUC, t1/2, MR |
| Park S et al. 2023 | Korea | All male | 29.1 | Healthy | RCT | 18 | Immediate and delayed-release formulations | Formulation | Cmax, Tmax, AUC, t1/2 |
| Yang E et al. 2023 | Korea | All male | 32.63 | Healthy | RCT | 19 | Proguanil Vs. Tegoprazan + Proguanil Vs. Esomeprazole + Proguanil Vs. Vonoprazan + Proguanil | DDI | Cmax, Tmax, AUC, t1/2, CL/F, fe, MR |
| Du Y et al. 2024 | China | Male : Female = 18 : 4 | 27.1 | Healthy | Non-RCT | 22 | Tegoprazan Vs. Tegoprazan + Bismuth + Amoxicillin + Clarithromycin  Bismuth + Amoxicillin + Clarithromycin Vs. Tegoprazan + Bismuth + Amoxicillin + Clarithromycin | DDI | Cmax, Tmax, AUC, Cmin |
| Kim HS et al. 2024 | Korea | All male | 28.34 | Healthy | RCT | 52 | Nasogastric tube Vs. oral dosing | Formulation | Cmax, Tmax, AUC, t1/2, CL/F, Vz/F |
| Fexuprazan: | | | | | | | | | |
| Sunwoo J et al. 2018 | Korea | All male | 29 | Healthy | Non-RCT | 8 | Fed Vs. Fasted | Food effect | Cmax, Tmax, AUC, t1/2, CL/F, CLr, fe, Rac |
| Hwang JG et al. 2020 | Korea | Male : Female = 66 : 13 | 32.37 | Healthy | RCT | 30 (Koreans)  19 (Caucasians)  30 (Japanese) | Koreans Vs.Caucasians Vs. Japanese | Ethnicity | AUC |
| Oh J et al. 2023 | Korea | Male : Female = 24 : 3 | 30.59 | Healthy | RCT | 27 | Fexuprazan Vs. Fexuprazan + Aspirin  Aspirin Vs. Fexuprazan + Aspirin | DDI | Cmax, Tmax, AUC, t1/2, MR |
| Shin W et al. 2023 | Korea | Male : Female = 19 : 5 | 27.17 | Healthy | RCT | 24 | 4 X 10 mg tablets Vs. 40 mg tablet | Formulation | Cmax, Tmax, AUC, t1/2, CL/F, Vz/F |
| Yang AY et al.2023 | Korea | Male : Female = 23 : 1 | 26.46 | Healthy | RCT | 24 | Size-reduced 20 mg tablet | Formulation | Cmax, Tmax, AUC, t1/2, CL/F, Vz/F |
| Won H et al. 2024 | Korea | All male | 28.3 | Healthy | RCT | 111 | Fexuprazan Vs. Fexuprazan + Celecoxib  Celecoxib Vs. Fexuprazan + Celecoxib  Fexuprazan Vs. Fexuprazan + Naproxen  Naproxen Vs. Fexuprazan + Naproxen  Fexuprazan Vs. Fexuprazan + Meloxicam  Meloxicam Vs. Fexuprazan + Meloxicam | DDI | Cmax, Tmax, AUC, t1/2, CL/F, Vz/F, MR |
| Keverprazan: | | | | | | | | | |
| Zhou S et al. 2023 | China | All male | Keverprazan 5 mg: 27.75 Keverprazan 10 mg: 24.88 Keverprazan 20 mg: 24.88 Keverprazan 40 mg: 27.75 Keverprazan 60 mg: 29.13 Lansoprazole 30 mg: 27.5 Placebo: 24.88 | Healthy | RCT | Keverprazan 5 mg (8)  Keverprazan 10 mg (8)  Keverprazan 20 mg (8)  Keverprazan 40 mg (8)  Keverprazan 60 mg (8)  Placebo10-60 mg (8)  Lansoprazole 30mg (8) | Keverprazan Vs. Lansoprazole | Direct comparison | Cmax, Tmax, AUC, t1/2, CL/F, CLr, Vz/F, MR |
|  |  |  | Fasted: 26.57 Fed: 28.57 | Healthy | Non-RCT | 14 | Fed Vs. Fasted | Food effect | Cmax, Tmax, AUC, t1/2, CL/F, Vz/F, MR |
| Zhou S et al. 2023 | China | Male : Female = 12 : 14 | Keverprazan: 22.63 Vonoprazan: 26.00 | Healthy | RCT | 8 (Keverprazan)  4 (Vonoprazan ) | Keverprazan Vs. Vonoprazan | Direct Comparison | Cmax, Tmax, AUC, t1/2, CL/F, Vz/F, MR, Tmin, Cmin, CAVSS, DF, Rac |
| Zastaprazan: | | | | | | | | | |
| Hwang I et al. 2023 | Korea | All male | 27.5 | Healthy | RCT | 8 | Fed Vs. Fasted | Food effect | Cmax, Tmax, AUC, t1/2, CL/F, CLr, Fe, Rac |
| Abbreviations: Aeτ, amount of drug excreted in the urine during a dosing interval; AUC, area under the concentration curve; CAVSS, average value of the steady-­state concentration; CL/F, apparent clearance; CLr, renal clearance; Cmax, peak concentration; Cmin, trough concentration; DF, degree of fluctuation; Fe, fraction of drug excreted unchanged in urine; λz, elimination rate constant; MR, metabolic ratio; MRT, mean resident time; Rac: accumulation ratio; t1/2, elimination half-­life; Tmax, time to reach Cmax; Tmin, time to reach Cmin; Vz/F, apparent volume of distribution; RCT, Randomized Controlled Trial. | | | | | | | | | |
